# Hypoxia‐activated ADCC‐enhanced humanized anti‐CD147 antibody for liver cancer imaging and targeted therapy with improved selectivity

**DOI:** 10.1002/mco2.512

**Published:** 2024-03-11

**Authors:** Fang‐Zheng Qi, Hui‐Shan Su, Bo Wang, Luo‐Meng Qian, Yang Wang, Chen‐Hui Wang, Ya‐Xin Hou, Ping Chen, Qing Zhang, Dong‐Mei Li, Hao Tang, Jian‐Li Jiang, Hui‐Jie Bian, Zhi‐Nan Chen, Si‐He Zhang

**Affiliations:** ^1^ Department of Cell Biology, School of Medicine Nankai University Tianjin China; ^2^ National Clinical Research Center for Cancer Tianjin Medical University Cancer Institute and Hospital Tianjin China; ^3^ State Key Laboratory of Medicinal Chemical Biology, College of Pharmacy and Tianjin Key Laboratory of Molecular Drug Research Nankai University Tianjin China; ^4^ National Translational Science Center for Molecular Medicine, Department of Cell Biology State Key Laboratory of Cancer Biology Air Force Medical University Xi'an China

**Keywords:** cluster of differentiation 147, hypoxia activation, liver cancer, on‐target off‐tumor toxicity, therapeutic antibody

## Abstract

Therapeutic antibodies (Abs) improve the clinical outcome of cancer patients. However, on‐target off‐tumor toxicity limits Ab‐based therapeutics. Cluster of differentiation 147 (CD147) is a tumor‐associated membrane antigen overexpressed in cancer cells. Ab‐based drugs targeting CD147 have achieved inadequate clinical benefits for liver cancer due to side effects. Here, by using glycoengineering and hypoxia‐activation strategies, we developed a conditional Ab‐dependent cellular cytotoxicity (ADCC)‐enhanced humanized anti‐CD147 Ab, HcHAb18‐azo‐PEG_5000_ (HAP18). Afucosylated ADCC‐enhanced HcHAb18 Ab was produced by a fed‐batch cell culture system. Azobenzene (Azo)‐linked PEG_5000_ conjugation endowed HAP18 Ab with features of hypoxia‐responsive delivery and selective targeting. HAP18 Ab potently inhibits the migration, invasion, and matrix metalloproteinase secretion, triggers the cytotoxicity and apoptosis of cancer cells, and induces ADCC, complement‐dependent cytotoxicity, and Ab‐dependent cellular phagocytosis under hypoxia. In xenograft mouse models, HAP18 Ab selectively targets hypoxic liver cancer tissues but not normal organs or tissues, and has potent tumor‐inhibiting effects. HAP18 Ab caused negligible side effects and exhibited superior pharmacokinetics compared to those of parent HcHAb18 Ab. The hypoxia‐activated ADCC‐enhanced humanized HAP18 Ab safely confers therapeutic efficacy against liver cancer with improved selectivity. This study highlights that hypoxia activation is a promising strategy for improving the tumor targeting potential of anti‐CD147 Ab drugs.

## Graphical abstract

Hypoxia‐activated HAP18 Ab was designed by conjugating HcHAb18 Ab with PEG_5000_‐azo. HAP18 Ab comprises target‐recognized unit controlled by caging moiety such that tethered PEG_5000_ could block its binding with CD147 antigen. Once azo‐bond was reduced under hypoxia, a self‐immolative reaction releases PEG_5000_, and HAP18 Ab is allowed to target CD147 in hypoxic liver cancer, thus eradicating the on‐target off‐tumor toxicity.

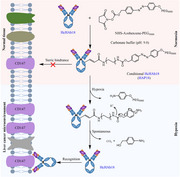

## 1 INTRODUCTION

Therapeutic antibodies (Abs) that target tumor‐associated membrane antigen (TAMA) have been prosperously developed. Most TAMAs are highly expressed on cancer cells, not exclusively.^1^ Therefore, therapeutic Abs that target an antigen (Ag) on cancer cells can also recognize the same Ag on normal cells, resulting in on‐target off‐tumor toxicity. Cluster of differentiation 147 (CD147) is a highly expressed TAMA that promotes the malignancy of liver cancer through multiple mechanisms. CD147 stimulates the production of matrix metalloproteinases (MMPs) in stromal fibroblasts, endothelial cells, and cancer cells.^2^, ^3^ These characteristics make CD147 a new target for Ab targeting. Metuximab (HAb18) is the first‐generation Ab drug that targets CD147. It benefits to liver cancer patients after orthotopic liver transplantation or in combination with radiofrequency ablation.^4^, ^5^ Metuzumab (HcHAb18), a second‐generation drug, is a chimeric anti‐CD147 Ab.^6^, ^7^, ^8^ In clinical trials, Metuzumab demonstrated inadequate antitumor efficacy in cancer patients (CTR20150433). This is largely attributed to the side effects such as rash, hemagglutination, and chills.^2^, ^3^, ^4^, ^5^, ^6^, ^7^, ^9^, ^10^, ^11^, ^12^ These side effects are derived from the residual low‐level expression of CD147 in some normal tissues,^3^, ^13^, ^14^ standing out CD147‐targeting therapies are not spatially restricted to cancer tissue, and peripheral toxicity may reduce the therapeutic efficacy.

To overcome the side effects in developing Metuzumab‐based therapies, a passive strategy is to reduce the dose to ameliorate systemic toxicity. However, clinical activity of HcHAb18 Ab at the tolerated dose decreased. One active delivery strategy is improving the targeting selectivity of HcHAb18 Ab based on tumor microenvironment. Solid tumors present unique microenvironments, such as high secretion of enzymes, low pH, and hypoxia. These microenvironmental features are distinct from those of normal tissues and can trigger specific chemical reactions. Hypoxic tumor microenvironment has been exploited to design stimuli‐responsive prodrugs or nanodrugs.^15^, ^16^, ^17^, ^18^ Hypoxic cells produce 100–1000 times more nitroreductase than normoxic cells, which can reduce azobenzene (azo)‐containing drugs for conditional release.^19^, ^20^, ^21^ Polyethylene glycol (PEG), a medical excipient, has excellent biocompatibility and resistance to nonspecific absorption. Drugs conjugated with PEG, known as PEGylation, could improve circulation stability and drug availability.^22^ Thus, we hypothesize that if azo‐linked PEG was conjugated to HcHAb18 Ab, the resulting HcHAb18‐azo‐PEG_5000_ (HAP18 Ab) could precisely target hypoxic liver cancer with improved selectivity.

**FIGURE 1:**
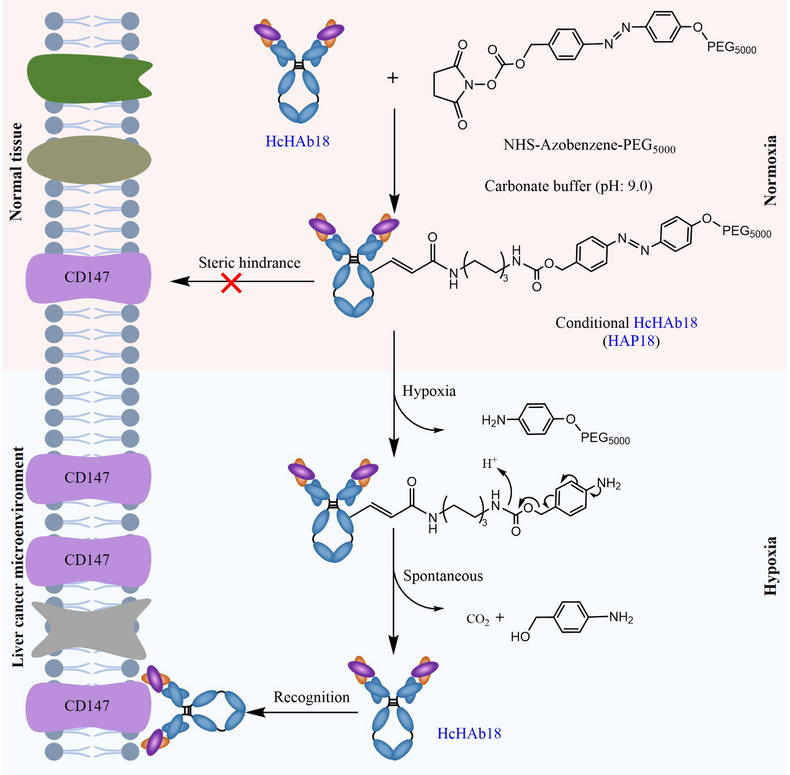
Schematic diagram illustrating the targeted therapy of hypoxia‐activated humanized HAP18 Ab.

In this study, a hypoxia‐activated HAP18 Ab was designed by conjugating HcHAb18 Ab with PEG_5000_‐azobenzene‐N‐hydroxysuccinimidyl (PEG_5000_‐azo‐NHS) ester. This conditional Ab comprises a target‐recognized unit controlled by a caging moiety such that tethered PEG_5000_‐azo sterically blocks parent Ab binding to CD147 Ag. Once the azo‐bond was reduced under hypoxia, the reductive cleavage of azo groups yields a self‐immolative aminobenzyl carbamate that releases lysine and 4‐aminobenzyl alcohol, a byproduct with little cytotoxicity.^19^, ^20^, ^21^ Upon recovery of intact structure initiated by the 1,6‐elimination reaction,^13^, ^14^ PEG_5000_ is released, and conditional HAP18 Ab is allowed to specifically target CD147 on hypoxic cancer cells. Thus, eradicating the on‐target off‐tumor toxicity for liver cancer therapy (Figure 1).

## 2 RESULTS

### 2.1 Development of an Ab‐dependent cellular cytotoxicity‐enhanced HAP18 Ab

**FIGURE 2:**
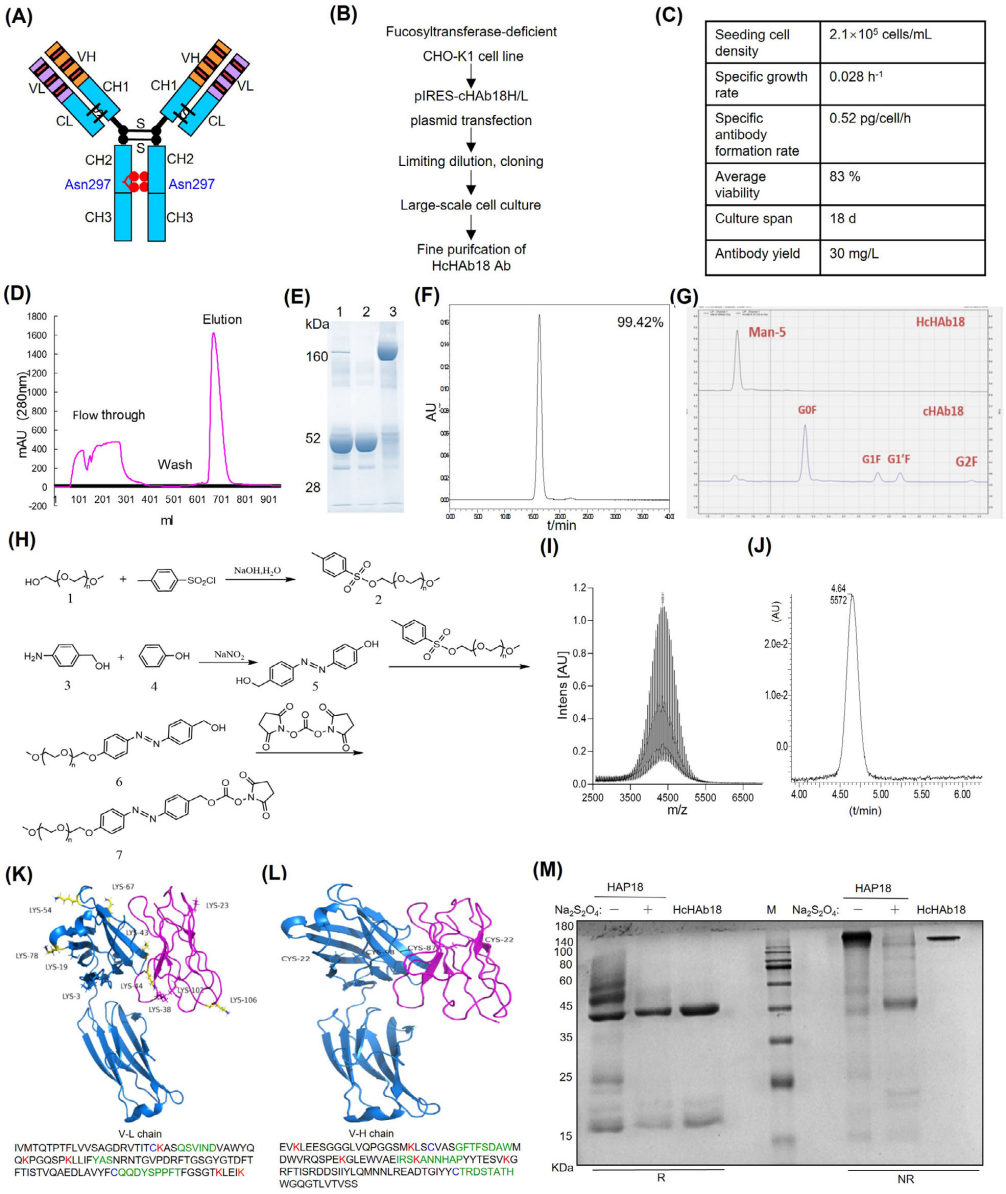
Development of conditionally activated HAP18 Ab. (A) Diagram of developing Ab‐dependent cellular cytotoxicity (ADCC)‐enhanced humanized HcHAb18 Ab. (B) Roadmap for producing defucosylated HcHAb18 Ab. After random mutagenesis induced by ICR‐191 and ethyl methanesulfonate (EMS), limiting dilution, and Lens culinaris agglutinin (LCA)‐resistance‐based cell cloning, CHO‐K1 cell lines deficient in a 1,6‐fucosyltransferase (FUT8), the only enzyme capable of Ab fucosylation in native cells, were selected. The HcHAb18 Ab was expressed in FUT8‐deficient CHO cells which produced defucosylated Ab. (C) Parameters of HcHAb18 expressing FUT8‐deficient CHO cells cultured in fed‐batch mode with 50 L of serum‐free medium. (D) Cation‐exchange chromatography analysis of HcHAb18 Ab captured by STREAMLINE expanded bed adsorption. A 510 cm inner diameter settled bed height Streamline 50 column was used. (E) SDS–PAGE analysis of the fractions from sample application (1), washing (2), and elution (3) steps during expended bed absorption purification. (F) Size‐exclusion high‐performance liquid chromatography (SEC‐HPLC) analysis of the purified HcHAb18 Ab. (G) Glycans were released from the Abs by PNGase F treatment and analyzed by high‐performance anion exchange chromatography with pulsed amperometric detection (HPAEC‐PAD). Glycans released from HcHAb18 (top) and cHAb18 (bottom) are shown. G0F, G1F, and G2F represent fucosylated biantennary complex‐type N‐glycans with 0, 1, and 2 terminal galactose residues, respectively; G1′F represents the fucosylated G1 positional isomer. (H) Synthesis scheme of the PEG_5000_‐azo‐NHS ester (compound #7). (I) Mass spectrum of the PEG_5000_‐azo‐NHS ester (compound #7). (J) HPLC absorbance spectrum of PEG_5000_‐azo‐NHS ester (compound #7). (K and L) The VL (magenta) and VH (blue) structures of HcHAb18 Ab were predicted by PIGS programs, and accessible lysine (yellow bond) residues are shown. In a head‐on view with respect to the idiotope rotated 90° (E), the VH and VL structures of HcHAb18 were rendered to show the surface feature that contributes to its idiotope by its complementarity determining regions (CDRs) (green characters as shown below). (M) SDS–PAGE analysis of HAP18 Ab with/without Na_2_S_2_O_4_ pretreatment. Ab samples were loaded under nonreducing and reducing conditions.

As glycoengineered Abs enhance the Fc‐effector function in cancer treatment,^23^ the fucosyltransferase‐deficient CHO‐K1 cell line was first developed through host cell glycoengineering technology (Eureka Therapeutics). Then, the humanized HcHAb18 Ab was expressed in this cell line, which generated afucosylated Abs with enhanced Ab‐dependent cellular cytotoxicity (ADCC) activity (Figure 2A,B). After serum‐free cell culture in a bioreactor with fed‐batch mode, the resulting ADCC‐enhanced HcHAb18 Ab was purified (Figure 2C–F). High‐performance anion exchange chromatography with pulsed amperometric detection‐based glycan profile analysis was performed to determine the Ab‐associated carbohydrate structure. Analysis of oligosaccharides released by PNGase F indicated that the chimeric HAb18 (cHAb18) Ab contained a mixture of G0, G1, and G2 carbohydrate structures with fucose attached. In contrast, the afucosylated HcHAb18 Ab contained less fucose and exhibited homogeneous glycosylation with the Man5 glycoform (Figure 2G). These results, together with previous reports,^6^, ^8^ suggest that afucosylated ADCC‐enhanced HcHAb18 Ab was successfully produced on a large scale.

To develop the conditional HAP18 Ab, an amine‐reactive PEG_5000_‐azo‐NHS ester was synthesized as a caging moiety according to previous reports with mini modifications (Figures 2H–J and S1–S4).^19^, ^24^, ^25^ Structural modeling of HcHAb18 Ab revealed that five and three lysine residues for PEGylation were surface accessible on the light chain variable region (VL) and hight chain variable region (VH) domains, respectively (Figure 2K,L and Table S1). After conjugating HcHAb18 Ab with as‐prepared PEG_5000_‐azo‐NHS, both reducing and nonreducing gel electrophoresis showed that HAP18 Ab with increased molecular weight were produced (Figure 2M). To mimic the hypoxia activation, reconversion reactions were performed by treating HAP18 Ab with sodium dithionite (Na_2_S_2_O_4_), which can cleave azo bonds.^19^, ^24^ Gel electrophoresis revealed that the bands corresponding to HAP18 Ab disappeared after Na_2_S_2_O_4_ treatment, whereas the bands corresponding to HcHAb18 Ab reappeared (Figure 2M). To determine the conjugation efficiency, 2,4,6‐trinitrobenzenesulfonic acid (TNBSA) assays were performed, and an average of 72% of the accessible lysine residues were azo‐conjugated with PEG_5000_. Again, Na_2_S_2_O_4_ treatment abolished this conjugation (Figure S5). Assessing the Ab pharmacokinetics showed that a single intravenous (i.v.) injection of parental Ab at low or high doses decreased the half‐life (*t*_1/2_) at 18 and 52 h, respectively. HAP18 Ab significantly extended the half‐life to 40 and 260 h, respectively (Figure S6). These results suggest that HAP18 Ab was successfully created and that PEG conjugation resulted it in a slow clearance rate.

### 2.2 Activated HAP18 Ab selectively targets CD147 Ag on hypoxic liver cancer cells

**FIGURE 3:**
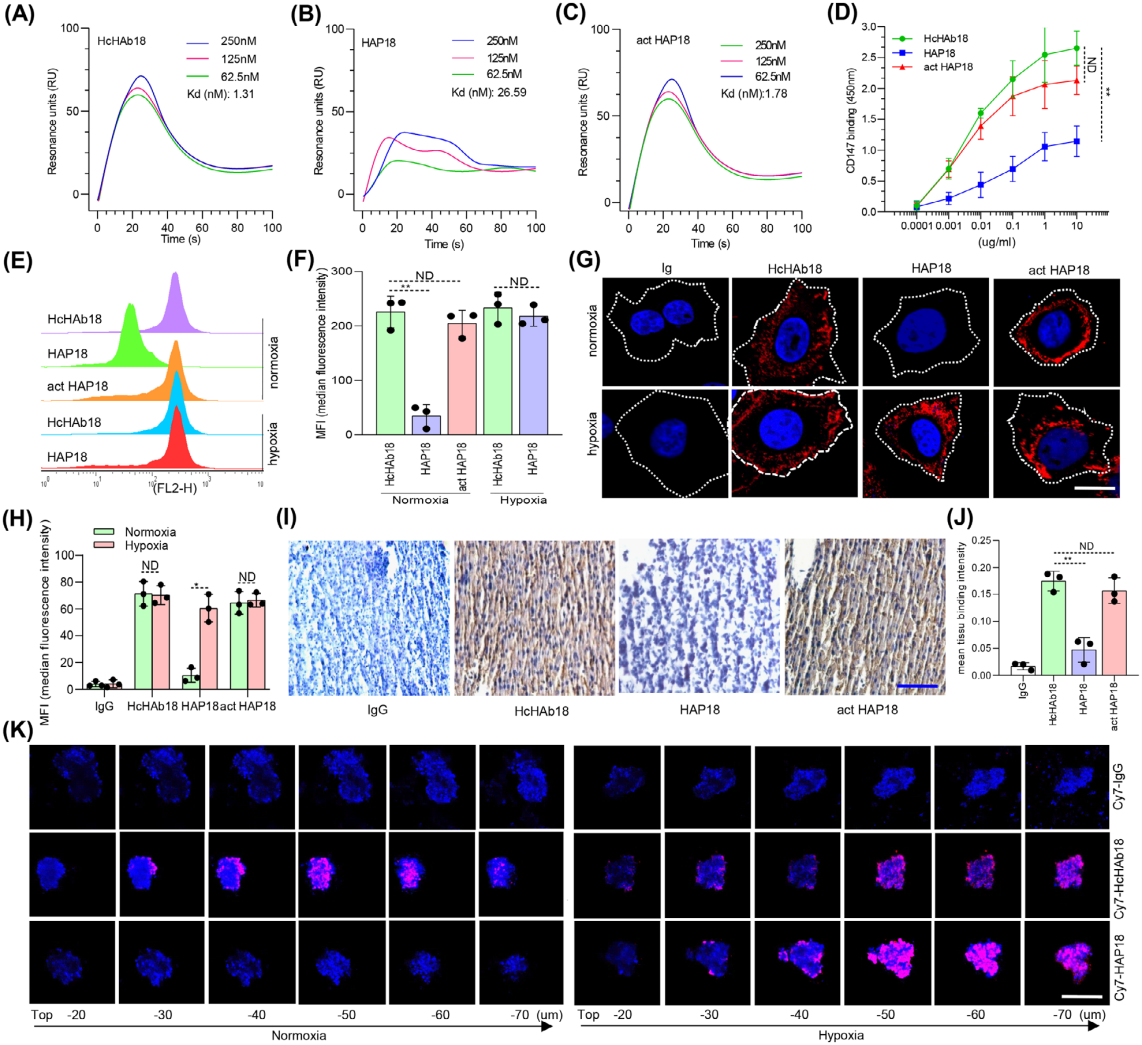
In vitro targeted delivery of the hypoxia‐activated HAP18 Ab. Surface plasmon resonance (SPR) determining the affinity of HcHAb18 (A), HAP18 (B), and Na_2_S_2_O_4_‐cleaved act HAP18 (C) Abs to CD147 Ag. (D) ELISA analysis of different Abs bound to CD147 Ag. *n* = 3. (E and F) Flow cytometry analysis of different Abs bound to HepG2 cells cultured under hypoxia or normoxia. Statistically difference compared with that of immunoglobulin G (IgG) (control) is shown (F). *n* = 3. (G and H) Confocal imaging of different Abs bound to HepG2 cells under hypoxia or normoxia. The Abs binding was quantified (H) and compared with that of IgG. Scale bar: 2 µm. *n* = 30. (I and J) Immunohistochemistry (IHC) staining of liver cancer samples from late‐stage liver cancer patients. Scale bar: 50 µm. Abs bound to liver cancer were quantified (J). IgG was used as a control. *n* = 10. (K) Confocal imaging of different Abs penetrating and bound to HepG2 MCTSs under hypoxia or normoxia. The fluorescence signal was collected at different levels from the top to the middle of spheroids on the *z*‐axis. Scale bar: 100 µm. *n* = 8. Representative data listed above are shown from at least two independent experiments. Significant differences compared with the control are shown. All the data are presented as mean ± standard deviation (SD). *p*‐Values (^*^ *p* ≤ 0.05, ^**^ *p* ≤ 0.01) were calculated two‐way via ANOVA and Student's *t* test.

To validate the state‐of‐the art design of conditional Abs, we tested the in vitro binding of HAP18 Ab to purified CD147 Ag. Surface plasmon resonance (SPR) results showed that the affinity of HAP18 Ab was 20 times lower than that of HcHAb18 Ab, and this attenuation was reversed when HAP18 Ab was cleaved by Na_2_S_2_O_4_ (Figure 3A–C). ELISA results showed that, compared with that of HcHAb18 Ab, the CD147 binding by HAP18 Ab was markedly decreased even at high concentrations, and Na_2_S_2_O_4_‐cleaved act HAP18 Ab completely recovered the binding to CD147 (Figure 3D). Next, we tested the cellular binding of HAP18 Ab. Flow cytometry results showed that HAP18 Ab did not recognize CD147 Ag on normoxia‐cultured liver cancer cells. However, Na_2_S_2_O_4_‐cleaved HAP18 Ab restored the binding to liver cancer cells. Such activation‐triggered binding recovery was observed when HAP18 Ab was added to hypoxic cancer cells (Figure 3E,F). Confocal imaging further showed that HAP18 Ab binding to liver cancer cells was abolished under normoxia. In contrast, its cellular binding was equivalent to that of HcHAb18 Ab when the cells were subjected to hypoxia. Additionally, Na_2_S_2_O_4_‐cleaved HAP18 Ab bound to normoxic and hypoxic cells in a similar manner (Figure 3G,H). These results suggest that PEG conjugation could cause steric hindrance to prevent HcHAb18 Ab from targeting CD147 under normoxia, but release such hindrance when hypoxia occurs. The binding of HAP18 Ab to liver cancer tissues was further checked. Compared with HcHAb18 Ab, as‐prepared HAP18 Ab barely bound to liver cancer tissues. However, once cleaved by Na_2_S_2_O_4_, it strongly bound to cancer tissue (Figure 3I,J). Multicellular tumor spheroids (MCTSs) have been proposed as models for evaluating hypoxia‐activated drug delivery.^24^ We prepared HepG2 MCTSs as models to assess whether HAP18 Ab could recognize CD147 Ag in hypoxic liver cancer. Normoxia‐ and hypoxia‐cultured HepG2 MCTSs were incubated with Cy7‐labeled HAP18 Ab or controls (act HAP18, HcHAb18, and immunoglobulin G [IgG]). The Ab‐directed fluorescence signal in MCTSs was monitored by confocal imaging. In normoxia‐cultured MCTSs, treatment with Cy7‐labeled HcHAb18 Ab produced a clear penetrating fluorescence signal. However, no fluorescence signal was detected in normoxic MCTSs treated with Cy7‐labeled HAP18 Ab or IgG. Notably, in hypoxia‐cultured MCTSs, treatment with HAP18 Ab produced an intense penetrating fluorescence signal similar to that observed with HcHAb18 Ab (Figures 3K and S7). These results indicate that PEG conjugation could obstruct HAP18 Ab binding to CD147 Ag in normoxic cancer tissues, completely eliminating its targeting before hypoxia‐triggered reactivation.

### 2.3 Activated HAP18 Ab induces potent immunity and antitumor activity in vitro

**FIGURE 4:**
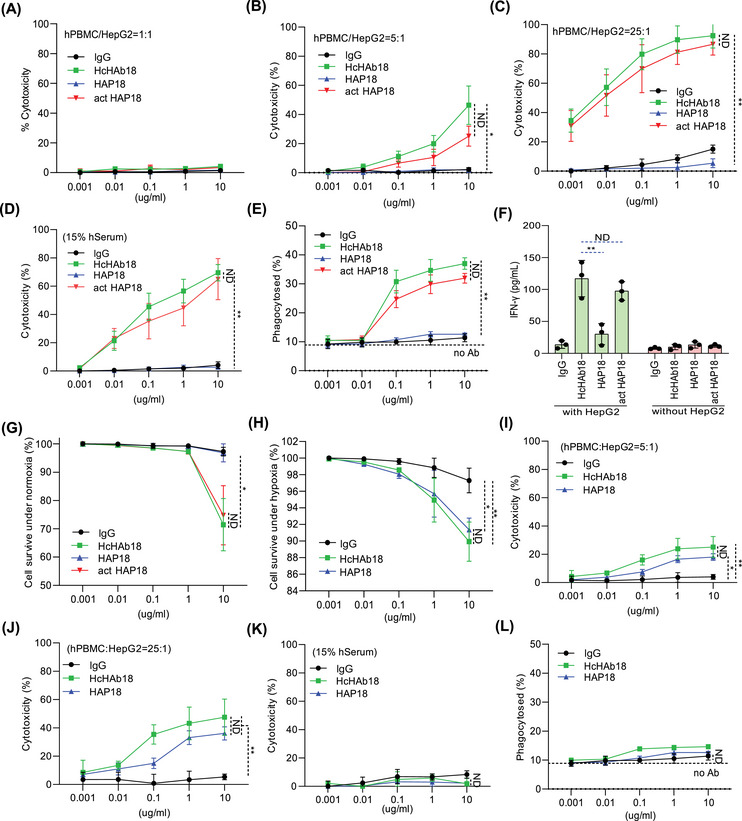
In vitro immunological killing of hypoxia‐activated HAP18 Ab. Ab‐dependent cellular cytotoxicity (ADCC) activity mediated by HcHAb18, HAP18, and act HAP18 Abs in LDH release assays. Peripheral blood mononuclear cells (PBMCs) were used as effectors, and HepG2 cells cultured under normoxia (A–C) or hypoxia (I and J) were used as targets at different effector/target ratios (A, 1:1; B and I, 5:1; C and J, 25:1). *n* = 3. (D and K) Complement‐dependent cytotoxicity (CDC) activity mediated by different Abs in HepG2 cells cultured under normoxia (D) or hypoxia (K). *n* = 3. (E and L) Ab‐dependent cellular phagocytosis (ADCP) activity mediated by Abs on macrophages was tested by flow cytometry. Normoxic (E) or hypoxic (L) HepG2 cells were used as targets. *n* = 3. (F) Interferon‐γ (IFN‐γ) secretion from Ab‐treated effector cells (hPBMCs) with/without target cells (HepG2) was determined via ELISA. *n* = 3. (G and H) Proliferation efficiency of normoxic (G) or hypoxic (H) HepG2 cells inhibited by different Abs was tested by CCK‐8 assay. *n* = 3. All the data are presented as mean ± standard deviation (SD). *p*‐Values (^*^ *p* ≤ 0.05, ^**^ *p* ≤ 0.01) were calculated via one‐way ANOVA and two‐way ANOVA.

**FIGURE 5:**
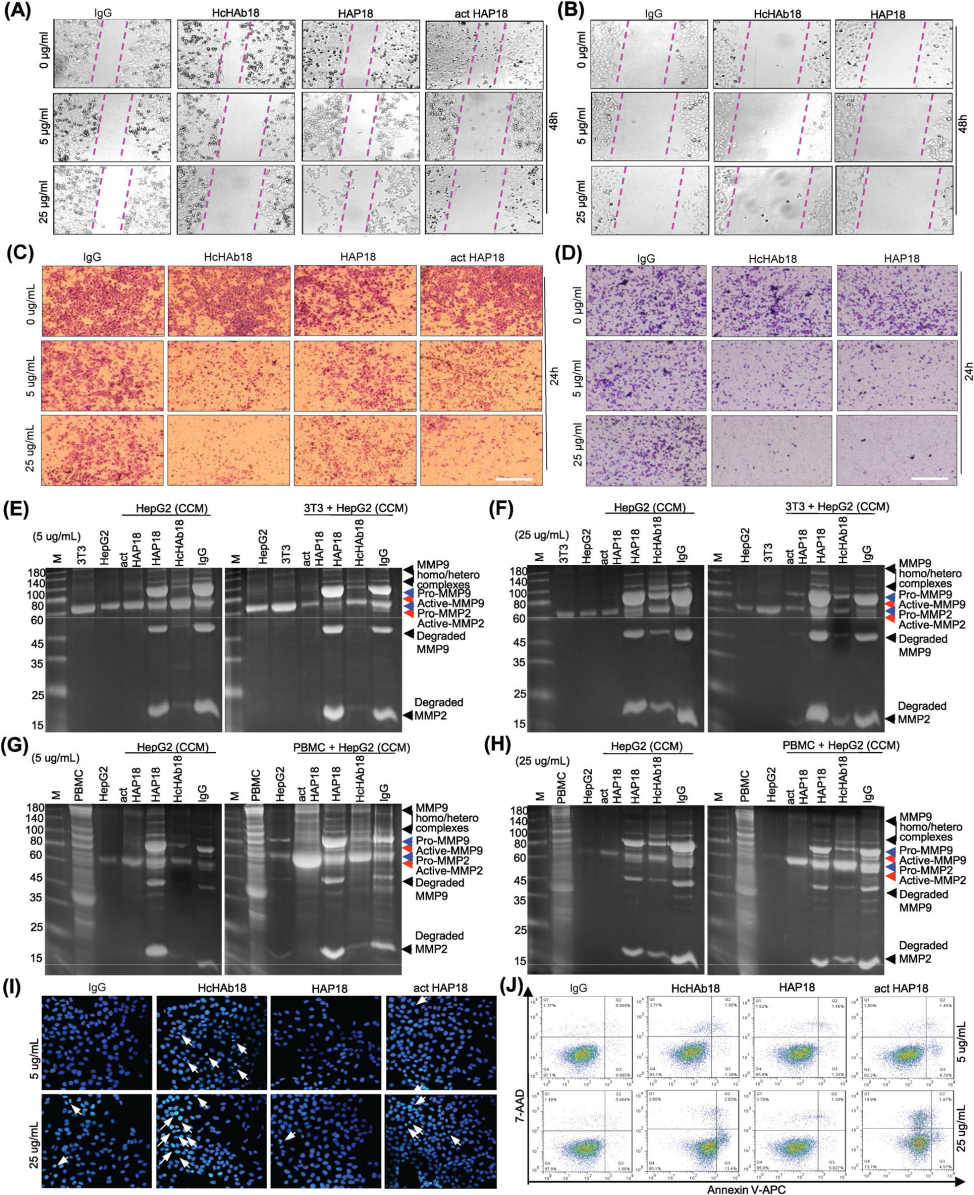
In vitro biological activity of the hypoxia‐activated HAP18 Ab. (A and B) Motility inhibition by different Abs in normoxic (A) or hypoxic (B) HepG2 cells was tested by scratch‐migration assays. *n* = 3. (C and D) Invasion efficiency of different Abs on normoxic (C) or hypoxic (D) HepG2 cells was tested by Matrigel‐coated transwell assays. Scale bars: 20 µm. *n* = 3. (E–H) Gelatin zymography was used to determine the activity of secreted MMPs in the concentrated culture medium (CCM) after the cells were exposed to different Abs for 24 h. Monoculture of HepG2 cells and coculture of HepG2 cells with 3T3 cells (E and F) or with hPBMCs (G and H) are presented. *n* = 2. (I) Hoechst 33342 staining of apoptotic HepG2 cells treated with different Abs. Apoptotic cell nuclei were deeply stained blue, and were dense and fragmented (marked with arrows). Scale bars: 20 µm. *n* = 3. (J) Flow cytometry was used to determine the degree of apoptosis in HepG2 cells triggered by different Abs for 24 h. Immunoglobulin G (IgG) was used as a control. *n* = 3. The representative data listed above are from at least two independent experiments. IgG was used as a control.

To test whether conditional HAP18 Ab could retain Fc‐effector functions, the ADCC activity of Abs was first analyzed. Human peripheral blood mononuclear cells (hPBMCs) were used as effectors and liver cancer cells were used as targets. Lactate dehydrogenase (LDH) release assays revealed that, under normoxia, HAP18 Ab completely lost its ADCC activity even when the effector/target ratio was increased to 25:1. Excitingly, such impaired ADCC activity was completely recovered when HAP18 Ab was activated by Na_2_S_2_O_4_ treatment (Figures 4A–C and S8A–C). We next checked whether the complement‐dependent cytotoxicity (CDC) activity of Ab was altered after conjugation with PEG_5000_. When fresh human serum (hSerum) was used as a complement source, LDH release results showed that the CDC activity of HAP18 Ab was completely lost. In contrast, Na_2_S_2_O_4_‐activated HAP18 Ab completely recovered its CDC activity (Figures 4D and S8D). We further tested whether the Ab‐dependent cellular phagocytosis (ADCP) activity of Ab was changed. Cellular phagocytosis assays revealed that, compared with HcHAb18 Ab, HAP18 Ab presented less ADCP activity. Again, the Na_2_S_2_O_4_‐activated HAP18 Ab restored the ADCP to a level similar to that of HcHAb18 Ab (Figures 4E and S8E). Interferon‐γ (IFN‐γ) released from NK cells is a necessary cytokine involved in immune‐mediated tumor killing. We cocultured hPBMCs (containing NK cells) with liver cancer cells and Abs. The supernatant was analyzed to determine the IFN‐γ level. ELISA results showed that HepG2 cells barely stimulated hPBMCs to release IFN‐γ, whereas HcHAb18 Ab treatment significantly enhanced the IFN‐γ release. In contrast, HAP18 Ab treatment produced little IFN‐γ, and the impaired IFN‐γ release was restored when HAP18 Ab was activated by Na_2_S_2_O_4_ (Figures 4F and S8F). These in vitro results suggest that selective targeting of HAP18 Ab can induce potent immunological responses. To test whether conditional HAP18 Ab could block CD147‐mediated malignant phenotypes, we further analyzed its antitumor activity at cellular level. Cell counting kit‐8 (CCK‐8) results showed that normoxic liver cancer cells treated with HAP18 Ab proliferated normally, whereas HAP18 Ab treatment markedly inhibited proliferation under hypoxia. This impaired proliferation was mimicked by incubating normoxic cells with Na_2_S_2_O_4_‐cleaved HAP18 Ab (Figures 4G,H and S8G,H). CD147‐promoted migration and invasion are essential for the malignancy of liver cancer.^26^, ^27^, ^28^ Scratch‐migration and Matrigel‐coated transwell assays showed that HAP18 Ab treatment, at either low or high concentrations, slightly inhibited the migration and invasion of liver cancer cells. However, Na_2_S_2_O_4_‐cleaved HAP18 Ab at both concentrations significantly inhibited migration and invasion, similar to the effects of HcHAb18 Ab (Figures 5A–D, S9A–D, and S10A–H). CD147‐mediated extracellular matrix (ECM)‐degradation is required for liver cancer invasion.^26^, ^27^, ^28^ Gelatin zymography results from mono‐ or cocultures showed that HAP18 Ab treatment did not attenuate MMP2 or MMP9 secretion. However, Na_2_S_2_O_4_‐cleaved HAP18 Ab significantly inhibited MMP2 and MMP9 secretion, which was similar to what was observed with HcHAb18 Ab. The difference in MMPs secretion was more obvious when the doses of HAP18 and activated HAP18 Abs were increased (Figures 5E–H and S11). As CD147 targeting can induce apoptosis in cancer cells,^29^ we further analyzed the Abs‐triggered apoptosis level. Hoechst and Annexin V/7‐AAD staining showed that HAP18 Ab treatment induced less apoptosis in liver cancer cells. However, Na_2_S_2_O_4_‐cleaved HAP18 Ab induced significant apoptosis, which was similar to that induced by HcHAb18 Ab (Figures 5I,J, S9E,F, and S10I–J). Similar results were observed for the apoptosis indicators by Western blotting (Figure S9G–H). These in vitro results suggest that HAP18 Ab induces effective antitumor activity by inhibiting migration, invasion, and MMP secretion and triggering apoptosis in liver cancer cells.

### 2.4 Activated HAP18 Ab exerts selective imaging and high therapeutic efficacy in vivo

**FIGURE 6:**
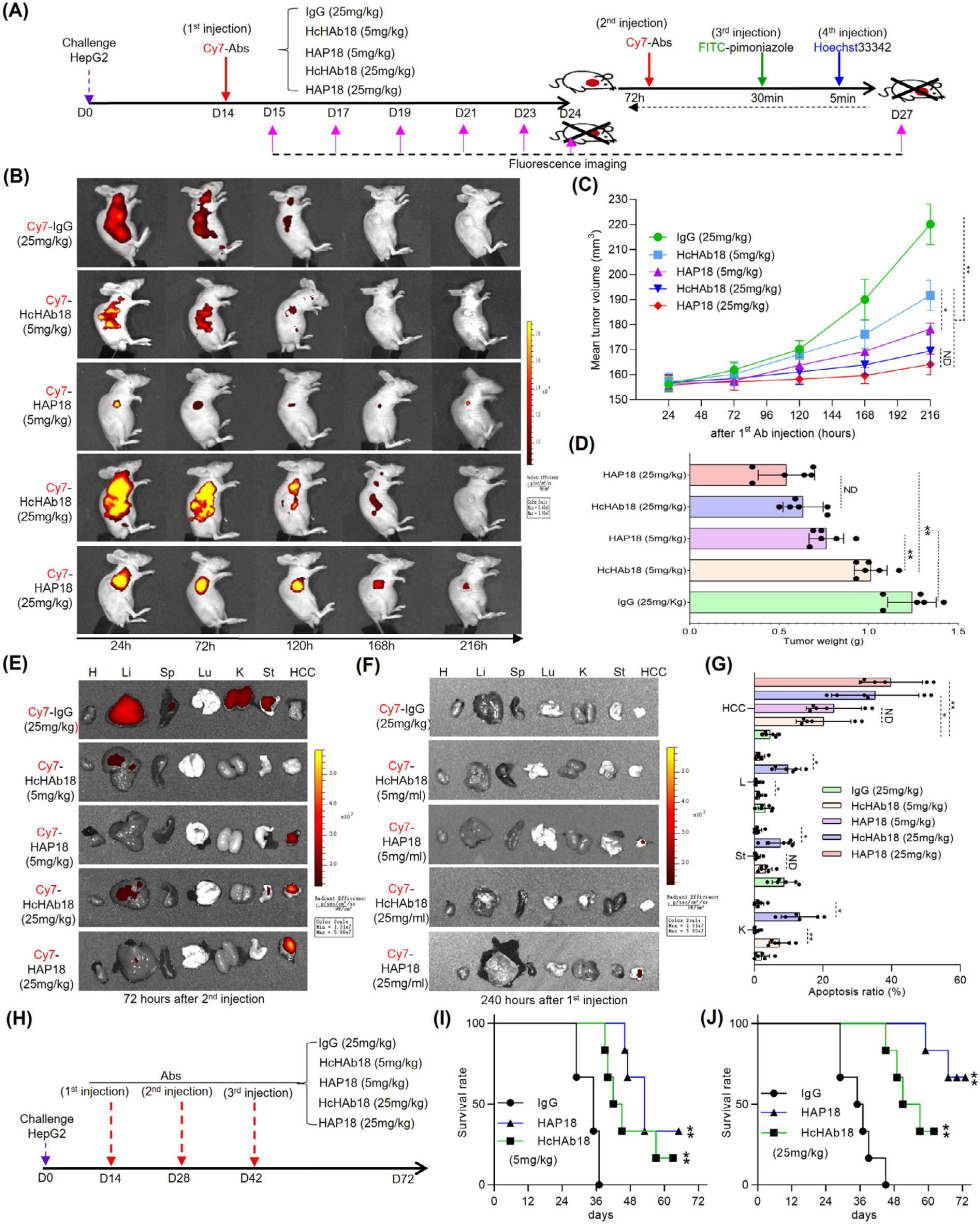
In vivo selective imaging and delivery of hypoxia‐activated HAP18 Ab for liver cancer targeted therapy. (A) Schedule of Ab imaging and therapy in human liver cancer‐xenografted mice model. To check the targeted selectivity, half of the mice (*n* = 6/group) received Abs (HcHAb18 and HAP18). Immunoglobulin G (IgG) was used as a control injection only once followed by dynamic fluorescence imaging. To check the hypoxia targeting, the other half of the mice (*n* = 6/group) received a second Ab injection followed by pimonidazole and Hoechst33342 injection for tissue‐section imaging. (B) Representative in vivo fluorescence imaging of tumor‐bearing mice injected with Cy7‐labeled Abs through the tail vein. (C) Growth curve of liver cancer volume during Ab treatment at low and high doses; bars represent the standard deviation (SD); *n* = 6/group. (D) Weights of excised tumors from the Ab‐treated groups; the bars represent the SDs; *n* = 6/group. (E and F) Representative ex vivo fluorescence signals from the main organs and liver cancer tissues of mice injected with Cy7‐labeled Abs through the tail vein. Heart (H), liver (Li), spleen (Sp), lung (Lu), kidney (K), stomach (St), and liver cancer tissues. *n* = 6/group. (G) Abs‐induced apoptotic signals from distinct tissues were quantified and analyzed; the bars represent the SDs; *n* = 6/group. (H) Schedule of HAP18 Ab‐based targeted therapy in liver cancer‐xenografted mice. (I and J) Survival rate after continuous Ab treatment (I: 5 mg/kg per mouse. J: 25 mg/kg per mouse) is illustrated by Kaplan–Meier curves. *n* = 8/group. All the data are presented as mean ± SD. *p*‐Values (^*^ *p* ≤ 0.05, ^**^ *p* ≤ 0.01) were calculated with Student's *t* test.

To test whether conditional HAP18 Ab could be used for in vivo tumor imaging, we systematically analyzed its ability to target tumors in nude mice xenografted with liver cancer cells (Figure 6A). As early as 24 h postinjection of Cy7‐labeled Abs through tail vein, fluorescence signals from the Abs accumulated at liver cancer sites. However, only HAP18 Ab, regardless of whether it was administered at a low or high dose, accumulated exclusively (Figure 6B). The liver cancer‐targeted fluorescence signal was constantly maintained for more than 9 days after the injection of Cy7‐labeled HAP18 Ab. In contrast, no accumulated contrast fluorescence signal was visualized in liver cancer postinjection of Cy7‐labeled HcHAb18 Ab, and the dispersal fluorescence signal disappeared between 120 and 168 h postinjection (Figure 6B). To analyze the targeting distribution of Abs, half of the mice in each group were sacrificed 240 h after the first injection, the other half were sacrificed 72 h after the second injection combined with pimonidazole, and the tumors and main organs were collected for fluorescence imaging. Ex vivo imaging showed that Cy7‐labeled HAP18 Ab selectively accumulated in xenografted liver cancer, whereas HcHAb18 Ab nonspecifically bound to liver and stomach. Such sharp‐contrasted targeting was more noticeable when the dose of Ab was increased or the duration of ex vivo imaging was extended (Figures 6E,F and S12). The targeting ability of conditional Ab was further analyzed by costaining excised cancer tissues with Abs against hypoxia inducible factor 1 alpha (HIF‐1α), microvascular marker CD31 and hypoxia marker pimonidazole. The signal from Cy7‐labeled HAP18 Ab, not Cy7‐labeled HcHAb18 Ab, was clearly colocalized with the signals from fluorescein isothiocyanate (FITC)‐labeled pimonidazole and AF488‐indicated HIF‐1α, but not overlapped with the signal from AF488‐indicated CD31 (Figure S13). These results suggest that HAP18 Ab could be selectively activated in vivo to recognize its Ag CD147 in hypoxic liver cancer tissues, whereas the parental Ab HcHAb18 could not. To test the therapeutic value of conditional Ab, the tumor‐killing efficacy in xenograft models was further analyzed. In the low‐dose groups (5 mg/kg), monotherapy with HAP18 Ab more significantly attenuated the progression of liver cancer, in terms of tumor volume and weight, than did treatment with HcHAb18 Ab (Figure 6C,D). TUNEL staining results showed that HAP18 Ab treatment significantly induced apoptosis in liver cancer tissues, which was equivalent to that induced by HcHAb18 Ab (Figures S14 and 6G). Notably, continuous i.v. injection of HAP18 Ab exhibited better tumor‐attenuation efficacy than treatment with HcHAb18 Ab in the high‐dose groups (25 mg/kg) (Figure 6H–J). Furthermore, three continuous HAP18 Ab injections yielded almost complete inhibition of tumor growth (Figure 6J). In contrast, repeated HcHAb18 Ab treatments only induced liver cancer regression in part mice. Importantly, compared with HcHAb18 Ab treatment, repeated HAP18 Ab injection significantly prolonged mice survival in both high‐ and low‐dose groups (Figure 6I,J). These results suggest that HAP18 Ab induced potent antitumor activity in vivo, which was vastly superior to that of HcHAb18 Ab.

### 2.5 Activated HAP18 Ab exerts improved biosafety without systemic toxicity in vivo

**FIGURE 7:**
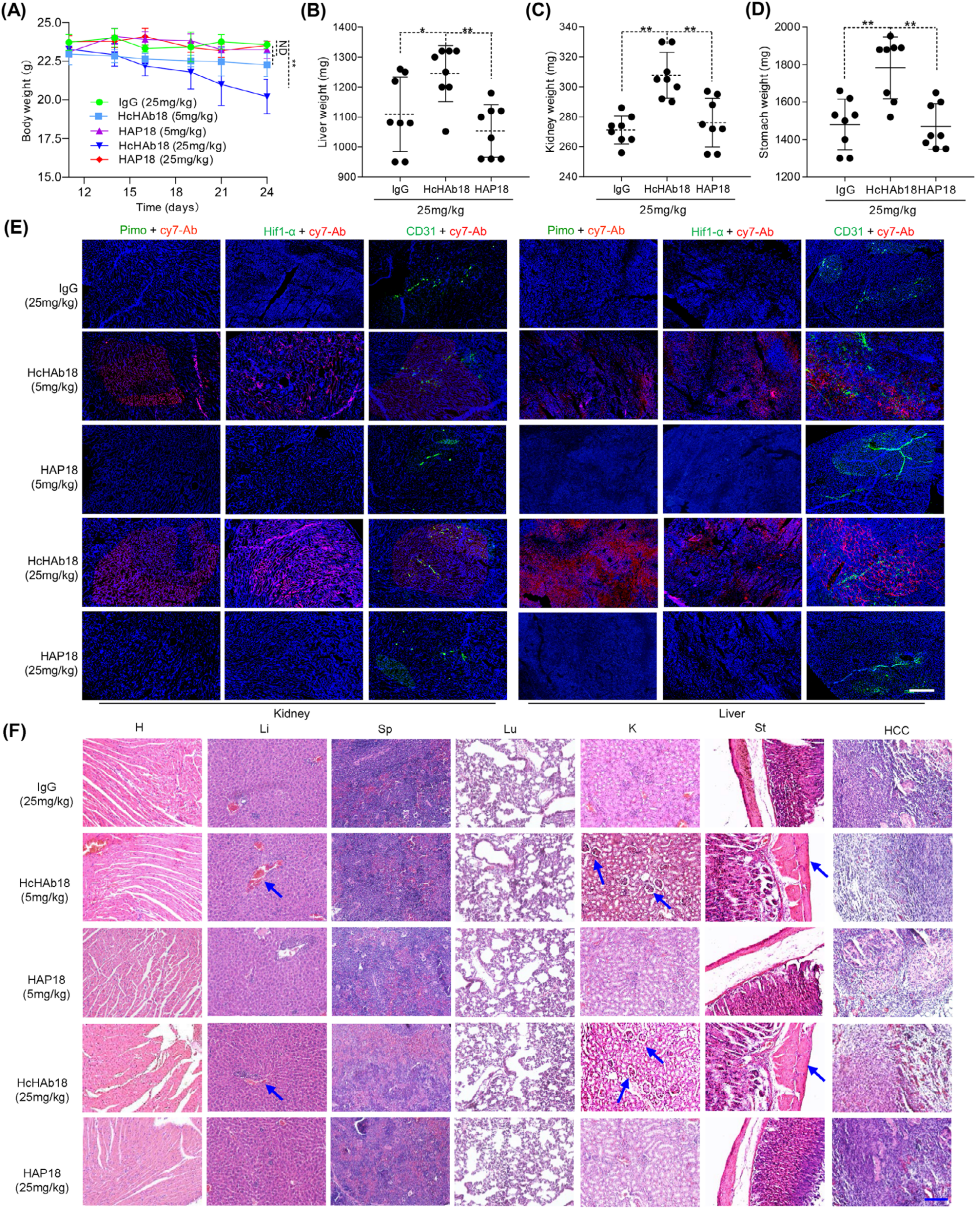
Ex vivo evaluation of the systemic toxicity of hypoxia‐activated HAP18 Ab. (A) Body weight curve of xenografted mice treated with different Abs (measured beginning on the 10th day postadministration with HepG2 cells); *n* = 8/group. (B–D) Liver, kidney, and stomach weights of mice treated with immunoglobulin G (IgG), HcHAb18, or HAP18 Abs. *n* = 8/group. All the data are presented as mean ± standard deviation (SD). *p*‐Values (^*^ *p* ≤ 0.05, ^**^ *p* ≤ 0.01) were calculated with Student's *t* test. (E) Representative colocalization signals of Cy7‐labeled Abs (red) with FITC‐pimonidazole (green), Hoechst 33342 (blue), or CD31 (green) in tissue sections of major organs from Ab‐treated mice. (F) Representative hematoxylin and eosin (H&E) staining of main organs and liver cancer tissues from Ab‐treated mice. Scale bar: 20 µm.

Finally, to investigate the safety of conditional HAP18 Ab, the toxicity profile of Abs in mice was investigated. No significant body weight loss was observed upon treatment with low or high dose of HAP18 Ab. In contrast, mice treated with high dose of HcHAb18 Ab experienced fluctuating weight loss (19–24 days) (Figure 7A). Checking the surgically resected organs from euthanized mice showed that treatment with HcHAb18 Ab at a high dose resulted in gross enlargement of the liver, kidney atrophy, and gastratrophia. In contrast, HAP18 Ab treatment barely resulted in hepatomegaly, kidney atrophy or gastratrophia as demonstrated by weight (Figure 7B,D). Diminished kidney toxicity and hepatotoxicity also mirrored the attenuated colocalized fluorescence signal between HAP18 Ab and pimonidazole or HIF‐1α in kidney and liver (Figure 7E). Hematoxylin and eosin staining of normal organs indicated that HAP18 Ab treatment caused no damage to major organs (Figure 7F). In contrast, treatment with HcHAb18 Ab caused significant mononuclear cell infiltration in liver, formed periportal cuffs with thickening of tunica media and infiltration foci associated with microvasculature, and a thickened stomach wall (Figure 7F). Mononuclear cell infiltration was also observed in lungs and kidney, where perivascular cuffs formed in mice treated with high dose of HcHAb18 Ab. These contrasting pathological changes were more obvious when the injected dose of Abs was increased. The effects of Abs on release of proinflammatory cytokines were also determined. Although INF‐γ level detected in serum was relatively low, a high dose of HcHAb18 Ab treatment triggered significant INF‐γ release. Similarly, HAP18 Ab treatment at high doses also induced substantial INF‐γ release (Figure S15). Together, these results suggested that conditional HAP18 Ab could alleviate systemic toxicity even at high doses.

## 3 DISCUSSION

Glycosylation is critical for Fc‐effector functions, including ADCC and CDC, which are major mechanisms of action by therapeutic Abs. Glycoengineering techniques have been evolved to develop stable and safe mAbs for high therapeutic efficacy.^30^ Here, we produced an aglycosylated HcHAb18 Ab in a large scale by using the host cell glycoengineering technique (Figure 2A–G). Determining the glycan profile of produced Abs revealed that fucosylated IgG form of cHAb18 Ab was secreted from wild‐type Chinese hamster ovary (CHO) cells. In contrast, the aglycosylated HcHAb18 Ab exhibited homogeneous glycosylation with a single major N‐glycan and a Man5 glycoform, but no fucose or xylose was detected. Such a glycoengineering modification enhances the ADCC activity of HcHAb18 Ab due to the absence of core fucose,^7^ and establishes the basis for superior tumor inhibition by HAP18 Ab (Figures 3, 4, 5, 6, 7 and S7–S15).

The liver is a typical hypervascular organ, whereas hypoxia is a common phenomenon in liver cancer. First, highly proliferating liver cancer cells induce local hypoxia when they are far from blood vessels. Second, most liver cancers develop through cirrhosis induced by liver injury. The latter causes fibrogenesis to demolish the liver blood system and leads to hypoxia.^18^, ^31^, ^32^, ^33^ Furthermore, palliative therapies for liver cancer always result in catastrophic hypoxia.^31^ Therefore, this hypoxic microenvironment can be capitalized to develop liver cancer‐selective targeted therapies. CD147 is a hub Ag that binds to other proteins (Intergrin, Hyaluronan, CD44, P‐gp/ABCB1, ABCG2, MCT‐1/4, CypA/B, and Gasdermin D) to drive the malignancy of cancer cells.^2^, ^3^, ^5^, ^14^, ^26^, ^27^, ^28^, ^34^ Previous studies revealed that hypoxia induces the upregulation of CD147 in liver cancer cells.^35^ Here, we found that hypoxia also increased CD147 expression on liver cancer cell surface (Figure S16). This affords a chance to target hypoxia‐upregulated CD147 via Abs,^3^ as most solid tumors are hypoxic. However, the ubiquitous expression of CD147 on nonmalignant cells, especially on blood cells and platelets, is a challenge. Compared with that of other hot targets, such as programmed cell death protein 1 (PD1), programmed cell death 1 ligand 1 (PDL1), and cytotoxic T lymphocyte‐associated protein 4 (CTLA4), the residual expression of CD147 in normal tissues is more obvious (Figure S17). Therefore, therapeutic Abs targeting CD147 on healthy cells may cause Ag sink and unwanted pharmacokinetics.^7^ Such on‐target off‐tumor toxicity can lead to clinical side effects resulting from anti‐CD147 Abs.

Most hypoxia‐responsive strategies are based on the use of quinones, nitroaromatics, and N‐oxides as release triggers. In contrast, the use of an azo moiety for hypoxia‐activated designs is relatively rare.^9^, ^21^, ^36^ Azo is a nitroimidazole derivative (R–N = N–R′), subject to oxygen‐dependent destabilization by reduction leading to cleavage of the linker. Moreover, azo is the substrate of azoreductase that is overexpressed in hypoxic solid tumors. Therefore, azo‐based molecules are sensitive to light, hypoxia, and certain enzymes, and hence show potential for use in site‐specific smart therapy.^37^ Some approaches use azo groups as scaffolds to conditionally quench imaging reagents, such as photosensitizer selenorosamine dyes,^38^ rhodamine derivatives,^20^, ^39^ and NIR probes.^36^, ^40^, ^41^ Other approaches incorporate an azo moiety in hypoxia‐degradable systems for conditional delivery of drug‐loaded nanomaterials.^9^, ^42^, ^43^ Several studies synthesized azo‐derivatives and conjugated them with chemotherapeutics.^33^, ^39^, ^44^, ^45^, ^46^ Only a few works, similar to our design (Figure 1), have adopted azo‐based conditional delivery of macromolecules. Prof. Tan's laboratory developed a hypoxia‐activated diagnostic probe by conjugating the aptamer with PEG‐azo.^24^ Although they also proposed the PEGylated conditional Ab, no data (especially in vivo) on therapeutic Ab drugs have been obtained. Prof. Zhong's laboratory has proposed hypoxia‐sensitive Ab–drug conjugates by conjugating monomethyl auristatin E (MMAE) to an anti‐HER2 Ab.^47^ Although tumor killing by such pro‐prodrugs is exciting, the cytotoxic effects are derived from hypoxia‐triggered MMAE release. From this perspective, our work is strongly supported by comprehensive in vitro and in vivo data (Figures 3, 4, 5, 6, 7 and S6–S15), highlighting that hypoxia‐activated Ab drugs can be feasibly developed by an azo‐based conjugation strategy.

One may argue that PEG‐azo was not site‐specifically conjugated to HAP18 Ab, and may lead to heterogeneous production. Whether site‐specific lysine conjugation is applicable depends on the application of HAP18 Ab. For developing ADCs, this approach may not be the best choice. However, in the case of developing stimulus‐activated Abs, saturated conjugation with PEG‐azo polymers (Figure S5C) reversibly disabled Ag binding by HAP18 Ab, as several lysine residues present in the Fab fragment are essential for the HcHAb18–CD147 interaction (Figure 2K,L). In addition, such azo‐PEGylation also increases the hydrodynamic size, extends the half‐life (Figures 6B–F and S6), and reduces immunotoxicity by masking HcHAb18 Ab from immune system. One may doubt that PEGylated Abs are large and unlikely to penetrate hypoxic cancer tissues. We observed the significant binding and penetration of HAP18 Ab in liver cancer MCTSs (Figure 3K) and observed similar penetration and targeting patterns in xenografted liver cancer (Figure S13). The penetration of therapeutic Abs into solid tumors depends on various factors, including dose, binding affinity, Ag expression level, tumor heterogeneity, and molecular size.^37^ PEGylation results in Abs with desirable properties such as increased hydrodynamic size, protection from proteolytic enzymes, and masked nonspecific recognition.^18^, ^32^ A low systemic clearance of PEGylated Abs with larger sizes is expected to increase tumor uptake by maintaining a greater diffusive gradient and allowing tumor uptake for a longer time.^32^, ^48^ In addition, due to the leaky vasculature that exists in solid tumors, passive targeting by Abs has been termed the “enhanced permeability and retention” effect.^17^, ^49^ Under this principle, PEGylated HAP18 Ab with increased size presumably exhibited enhanced targeting and accumulation in hypoxic liver cancer tissues.

Two points should be noted in this study. First, the immunological effects of HcHAb18 and HAP18 Abs were reduced under hypoxia. Ab‐induced effector functions include NK cell‐mediated ADCC, macrophage‐mediated ADCP, and CDC. Compared with the action under normoxia, these two Abs induced little CDC and ADCP and the triggered ADCC was diminished under hypoxia (Figures 4I–L and S8I–L). Such hypoxia‐impaired immunological effects are in line with previous observations that other Abs decrease the ADCC, ADCP, and CDC in tumors.^50^, ^51^, ^52^, ^53^, ^54^ The underlying mechanisms are unclear, but may be associated with hypoxia‐related downregulation of immune function.^51^, ^55^, ^56^ The decreased but not disappeared ADCC induced by HAP18 Ab is attributed to enhanced ADCC characteristic of HcHAb18 Ab (Figure 2). Second, compared with those of HcHAb18 Ab, a minor decrease in Ag binding and antitumor efficacy of HAP18 Ab was observed under hypoxia (Figures 3E–H and 6C,I,J). A minor decrease was also observed under normoxia when tested with act HAP18 Ab (Figures 3, 4, and S8–S12). Such minor decrease suggested that although Ab‐PEGylation provided desirable shield for removing nonspecific binding, its affinity to CD147 Ag also decreased slightly. This result is consistent with previous observations,^18^, ^48^ and site‐specific PEGylation may be considered in the future. Two limitations exist in this study. First, measuring immune responses on hPBMCs might not yield an accurate output. Since IFN‐γ produced in PBMCs can be induced by Ag stimulation or Ab targeting, detecting IFN‐γ levels via the current approach is acceptable. Further studies need to purify NK cells from hPBMCs and coculture them with liver cancer cells to precisely detect Ab‐triggered IFN‐γ release. Second, humanized HAP18 Abs bind to only cancer cells that express human CD147 in immunocompromised hosts. Thus, the antitumor efficacy of Abs may not be precisely estimated. Therefore, the hypoxia‐activating imaging and therapeutic advantages of HAP18 Ab should be further evaluated in immunocompetent hosts.

In summary, we developed a hypoxia‐activated ADCC‐enhanced HAP18 Ab by using glycoengineering and azo‐PEG‐conjugation strategies. This conditional Ab has three advantages: hypoxia‐dependent selective targeting to CD147 Ag; lacks HcHAb18 Ab‐associated toxicity; potential as immunotherapeutic drug and for enhancing liver cancer imaging and inhibition. This provides a promising strategy to alleviate the on‐target off‐tumor toxicity of Ab drugs, and promotes the use of hypoxia‐activated Abs for safe and effective precision therapy.

## 4 MATERIALS AND METHODS

### 4.1 Cell culture, blood sample, and main reagents

Human liver cell line HepG2, Huh7, and embryo fibroblast 3T3 obtained from the Type Culture Collection of the Chinese Academy of Sciences (China) were routinely cultured (fetal bovine serum, 10%) in normoxic (5% CO_2_) or hypoxic (O_2 _<1%) incubators. Human peripheral blood was collected from health examiners. PBMCs were isolated as described previously.^57^ The monocytes were collected in complete medium containing granulocyte macrophage‐colony stimulating factor (GM‐CSF) (50 ng/mL) and cultured for 1 week to generate the primary macrophages.

The anti‐CD147 chimeric Ab HcHAb18, mouse McAb HAb18, and CD147 Ag were originally engineered in our laboratory.^5^, ^6^, ^7^, ^8^, ^11^, ^26^, ^27^, ^28^, ^58^ Anti‐HIF‐1a (340462) and CD31 (347526) rabbit Abs were obtained from ZenBio. Rabbit anti‐pro‐caspase‐3 (04004), pro‐caspase‐9 (03421), cleaved caspase‐3 (01992), cleaved caspase‐9 (01838), and PARP (01932) Abs were obtained from Wanleibio. CCK‐8 (C0038), LDH cytotoxicity (C0016), and Annexin V‐APC/7‐AAD (KGA1026) kits were obtained from Beyotime. CM5 chips (BR100399) were obtained from Cytiva. Matrigel (0827015) was obtained from ABWbio. Human IFN‐γ ELISA kit (ml077386) was obtained from Mlbio. M‐CSF (91102ES08) was obtained from Yeasen. CFSE (B22022) was obtained from AAT Bioquest. PKH26 (HY‐D1451) was obtained from MedChemExpress. Cy7 dye (RH7775) was obtained from Ruixibio. FITC‐pimonidazole (HP2100) was obtained from Hypoxyprobe. PEG_5000_ (9004744) was obtained from Fchem. Na_2_S_2_O_4_ (7775146) was obtained from Adamas. TNBSA (2508192) and other chemicals were obtained from Sigma.

### 4.2 Synthesis of PEG_5000_‐azo‐NHS ester

PEG_5000_‐azo‐NHS ester was synthesized based on previous approaches.^19^, ^24^ Some modifications were made to the synthesis process by Thinkerytech Co. Ltd. The details are described in the Supporting Information.

### 4.3 Ab modeling and predicting accessible lysines

The canonical structure method was used to model HcHAb18 Ab.^59^ The accessible lysine residues on Ab surface were modeled to amine‐reactive PEG polymers by previous methods.^60^ The solvent‐accessible surface area (SASA) and accessible surface area were determined by using Amber 18 software (https://ambermd.org/). The SASA was estimated by using the approach of surf module with linear combination of pairwise overlaps.^61^ The complete surface area was determined by the molsurf module.

### 4.4 Generation, purification, and cleavage of HAP18 Ab

Excess PEG_5000_‐azo‐NHS ester and HcHAb18 Ab were reacted in carbonate buffer (0.1 M, pH 9.0). After overnight reaction (shaking, 200 rpm) at 4°C, the Ab was purified by Amicon Ultra centrifugal filter units. To activate the HAP18 Ab (act HAP18), equal volume of HAP18 solution (200 µM) was reacted with Na_2_S_2_O_4_ (100 µM) in carbonate buffer (pH 8.0) for 2 h at RT. After ultrafiltration, the activated samples were collected and aliquoted at −80°C for storage.

### 4.5 Pharmacokinetic analysis

Pharmacokinetic studies were conducted in female NOD mice for the parental HcHAb18 and HAP18 Abs. The details are described in the Supporting Information.

### 4.6 Coomassie blue staining and Western blotting

12% SDS‒PAGE gels were used to separate the purified Abs loaded with reducing or nonreducing buffer. After staining with Coomassie blue and washing, the gels were imaged and recorded with a Chemidox XRS system. The details of Western blotting are described in the Supporting Information.

### 4.7 Determination of Ab conjugation by TNBSA assay

Na_2_S_2_O_4_‐cleaved Abs (200 µg/mL) or 5‐amino‐1‐pentanol (20 µg/mL) was gradient diluted in NaHCO_3_ buffer (pH 8.5) and reacted with 0.1% (w/v) TNBSA solution for 2 h at 37°C. After adding with 10% SDS solution and HCl solution (1 N), the UV absorbance was measured with a microplate reader (335 nm). The contents of the primary reactive amines were determined by using the 5‐amino‐1‐pentanol‐based calibration curve.

### 4.8 Ab affinity, Ag binding, and IFN‐γ release assays

SPR experiments were conducted as previously described.^62^ All tests were performed by using a CM5 chip captured with human CD147 Ag. The details of Ab affinity, Ag binding, and IFN‐γ release assays are described in the Supporting Information.

### 4.9 Cellular and MCTSs binding assays

Liver cancer cells were cultured under hypoxia or normoxia for 24 h. EDTA‐detached cells were incubated with primary Abs and anti‐human IgG (H + L)‐RDM, then checked by flow cytometry and confocal imaging as previously described.^26^, ^27^, ^37^ MCTSs were prepared as previously described,^24^ and the MCTSs binding assays are described in the Supporting Information.

### 4.10 ADCC, CDC, and ADCP assays

ADCC was determined as previously described.^7^, ^10^ hPBMCs and liver cancer cells were used as effectors and targets, respectively. Different Abs (0.001–10 µg/mL) were incubated with normoxic or hypoxic target cells (1 × 10^4^ per well) at 37°C for 30 min, then the effector cells at different effector/target ratios (1:1, 5:1, 25:1) were added, and cultured for 48 h. LDH cytotoxicity kits were used to estimate cell viability. Treatment without PBMCs was defined as the negative control (NC) group. ADCC activity was calculated by using the equation: %ADCC = (*C* – *E*)/*C* × 100%, where *E* is the OD value from the experimental group and *C* is the fOD value of NC group. CDC was determined as previously described.^63^ Normoxic or hypoxic liver cancer cells were incubated with different Abs (0.001–10 µg/mL) at 37°C for 1 h. After the incubation, fresh hSerum from health examiners was used as a complement source and added to the culture (15% serum). After culture for 6 h, cell viability was analyzed by using LDH cytotoxicity kits. Heat‐inactivated serum was used as a NC. ADCP was assayed as previously described.^57^ Macrophages (2 × 10^5^ cells/mL) and liver cancer cells (4 × 10^4^ cells/mL) were labeled with CFSE and PKH26, respectively. The cells in medium contained different Abs (0.001–10 µg/mL) were cocultured (ratio, 5:1) under normoxia or hypoxia at 37°C for 6 h. Samples were analyzed by flow cytometry. Phagocytosis activity was quantified by using the equation: %phagocytosis = (1 – *T*)/TN, where *T* is the phagocytic events of in experimental group and TN is the phagocytic events of in NC group (without Ab). All assays were performed in triplicate, and the percentages of ADCC, CDC, and ADCP were plotted against the concentration of tested Ab.

### 4.11 In vitro cytotoxicity, growth inhibition, and apoptosis assays

These three assays were conducted under normoxic and hypoxic conditions as previously described.^27^, ^34^, ^64^, ^65^ The details are described in the Supporting Information.

### 4.12 Gelatin zymography, cell migration, and invasion assays

MMPs secretion, scratch migration, and matrigel‐coated transwell invasion assays were checked as previously described.^26^, ^27^, ^28^ The details are described in the Supporting Information.

### 4.13 In vivo and ex vivo imaging in xenografted mice model

Liver cancer cells (8 × 10^6^) were subcutaneously injected into female NOD mice. Liver cancer growth was monitored once a week by measuring the tumor volume. When the tumor volume reached 0.4 cm in diameter, the mice were randomly assigned to five main treatment groups (*n* = 12/group), and i.v. injected with Cy7‐labeled Abs (5 or 25 mg/kg) in phosphate‐buffered saline. Mice were imaged under anesthesia by an in vivo imaging system (IVIS 200, XENOGEN) at varied time points. The fluorescence intensity of images is recorded as photons per second per centimeter squared per steradian (p/s/cm^2^/sr).

For ex vivo imaging, Cy7‐labeled Abs (72 h before euthanization, i.v., 5 or 25 mg/kg), FITC‐pimonidazole (30 min before euthanization, intraperitoneal, 60 mg/kg), and Hoechst 33342 (5 min before euthanization, i.v., 1 mg/kg) were sequentially injected into half of the mice at allocated time points. After sacrificing the mice, liver cancers and livers, spleens, hearts, lungs, kidneys, and stomachs were dissected, the fluorescence signals from these organs and tumors were imaged and quantified.

### 4.14 In vivo therapy and toxicity study

To study the continuing therapeutic response, mice xenografted with liver cancer cells received three injections of naked Abs (5 or 25 mg/kg, *n* = 8/group). Liver cancer growth in mice was continuously monitored. Mice were euthanized when the tumors ulcerated, tumor size reached too large to burden, or when the sign of mouse distress observed. Cured and naive mice were followed for an additional 30 days after the last Ab challenge. One week after the last Ab therapy, the mice were killed, and the main organs were dissected, weighed, and fixed with 10% paraformaldehyde (PFA) for 48 h. The fixed tissues were washed, paraffin‐embedded, and the tissue sections were subjected for staining.

### 4.15 Immunofluorescence staining and immunohistochemistry

Dissected tissues were embedded in freezing medium (SAKURA) and snap frozen in liquid nitrogen. Tissue sections (10 µm) were fixed with ice‐cold acetone, rehydrated, blocked with 5% bovine serum albumin (BSA), double stained with CD31 (1:200) and HIF‐1α (1:200) Abs overnight, and incubated with goat anti‐rabbit IgG‐AF488 (1:1000). The distribution of multiple fluorescence signals was recorded under a fluorescence microscope.

Fresh surgical samples from liver cancer patients were collected from clinic. Immunohistochemistry (IHC) staining was performed as previously described.^26^, ^32^, ^34^, ^58^, ^64^, ^65^ Briefly, after deparaffinization, rehydration, and Ag retrieval with 10 mM sodium citrate buffer (pH 6.0) at 120°C, the slides were treated with hydrogen peroxide, blocked with goat serum, and overnight incubated with primary Abs at 4°C. Goat anti‐human IgG‐HRP was used as a secondary Ab. IHC staining was performed with an Envision two‐step system. The Ab‐based IHC staining signal was quantified by using ImageJ.

### 4.16 Statistical analysis

All the results are presented as mean ± standard deviation. Statistical analyses were executed with GraphPad Prism by using one‐ or two‐way ANOVA or Student's *t*‐test. Significant difference were defined as ^*^ *p *< 0.05 or ^**^ *p *< 0.01. Three independent assays were performed for each methodology unless otherwise stated.

## AUTHOR CONTRIBUTIONS

*Methodology, validation, formal analysis, and investigation*: F.‐Z.Q., H.‐S.S., and B.W. *Methodology, resources, and investigation*: L.‐M.Q., Y.W., C.‐H.W., and Y.‐X.H. *Resources and investigation*: P.C., Q.Z., D.‐M.L., and H.T. *Methodology, resources, and discussion*: J.‐L.J., H.‐J.B., and Z.N.C. *Conceptualization, supervision, project administration, funding acquisition, and writing—original draft and revision*: S.‐H.Z. All the authors have read and approved the final manuscript.

## CONFLICT OF INTEREST STATEMENT

The authors declare they have no conflicts of interest.

## ETHICS STATEMENT

All animal experiments were approved and supervised by Nankai University Animal Care and Use Committee (2021‐SYDWLL‐000479). Human blood samples were obtained from health examiners, and surgical specimens were obtained from liver cancer patients (informed consent was obtained from all participants, and approved by the Review Board of Nankai University, NKUIRB2021043).

## Supporting information

Supporting Information

## Data Availability

All data analyzed are included in this article and additional information is available upon request.

## References

[1] Beck A , Goetsch L , Dumontet C , Corvaïa N . Strategies and challenges for the next generation of antibody–drug conjugates. Nat Rev Drug Discov. 2017;16(5):315‐337. doi:10.1038/nrd.2016.268 28303026

[2] Huang D , Rao D , Jin Q , et al. Role of CD147 in the development and diagnosis of hepatocellular carcinoma. Front Immunol. 2023;14:1149931. doi:10.3389/fimmu.2023.1149931 37090718 PMC10115957

[3] Rahat MA . Mini‐review: can the metastatic cascade be inhibited by targeting CD147/EMMPRIN to prevent tumor recurrence? Front Immunol. 2022;13:855978. doi:10.3389/fimmu.2022.855978 35418981 PMC8995701

[4] Bian H , Zheng JS , Nan G , et al. Randomized trial of [131I] metuximab in treatment of hepatocellular carcinoma after percutaneous radiofrequency ablation. J Natl Cancer Inst. 2014;106(9):dju239. doi:10.1093/jnci/dju239 25210200

[5] Xu J , Shen ZY , Chen XG , et al. A randomized controlled trial of Licartin for preventing hepatoma recurrence after liver transplantation. Hepatology. 2007;45(2):269‐276. doi:10.1002/hep.21465 17256759

[6] Feng F , Wang B , Sun X , et al. Metuzumab enhanced chemosensitivity and apoptosis in non‐small cell lung carcinoma. Cancer Biol Ther. 2017;18(1):51‐62. doi:10.1080/15384047.2016.1276126 28055291 PMC5323017

[7] Zhang Z , Zhang Y , Sun Q , et al. Preclinical pharmacokinetics, tolerability, and pharmacodynamics of metuzumab, a novel CD147 human‐mouse chimeric and glycoengineered antibody. Mol Cancer Ther. 2015;14(1):162‐173. doi:10.1158/1535-7163.mct-14-0104 25376611

[8] Wang M , Zhang S , Sun Q , et al. Dual effects of an anti‐CD147 antibody for Esophageal cancer therapy. Cancer Biol Ther. 2019;20(12):1443‐1452. doi:10.1080/15384047.2019.1647052 31411555 PMC6804810

[9] Dong X , Mu L‐L , Liu X‐L , et al. Biomimetic, hypoxia‐responsive nanoparticles overcome residual chemoresistant leukemic cells with co‐targeting of therapy‐induced bone marrow niches. Adv Funct Mater. 2020;30(12):2000309. doi:10.1002/adfm.202000309

[10] Li Y , Zhang T , Pang Y , Li L , Chen ZN , Sun W . 3D bioprinting of hepatoma cells and application with microfluidics for pharmacodynamic test of Metuzumab. Biofabrication. 2019;11(3):034102. doi:10.1088/1758-5090/ab256c 31141796

[11] Wang Y , Yuan L , Yang XM , et al. A chimeric antibody targeting CD147 inhibits hepatocellular carcinoma cell motility via FAK–PI3K–Akt–Girdin signaling pathway. Clin Exp Metastasis. 2015;32(1):39‐53. doi:10.1007/s10585-014-9689-7 25424030

[12] Li J , Xing J , Yang Y , et al. Adjuvant (131)I‐metuximab for hepatocellular carcinoma after liver resection: a randomised, controlled, multicentre, open‐label, phase 2 trial. Lancet Gastroenterol Hepatol. 2020;5(6):548‐560. doi:10.1016/s2468-1253(19)30422-4 32164877

[13] Chen Y , Xu J , Wu X , et al. CD147 regulates antitumor CD8(+) T‐cell responses to facilitate tumor‐immune escape. Cell Mol Immunol. 2021;18(8):1995‐2009. doi:10.1038/s41423-020-00570-y 33177695 PMC8322173

[14] Asgari R , Vaisi‐Raygani A , Aleagha MSE , Mohammadi P , Bakhtiari M , Arghiani N . CD147 and MMPs as key factors in physiological and pathological processes. Biomed Pharmacother. 2023;157:113983. doi:10.1016/j.biopha.2022.113983 36370522

[15] Im S , Lee J , Park D , Park A , Kim YM , Kim WJ . Hypoxia‐triggered transforming immunomodulator for cancer immunotherapy via photodynamically enhanced antigen presentation of dendritic cell. ACS Nano. 2019;13(1):476‐488. doi:10.1021/acsnano.8b07045 30563320

[16] Perche F , Biswas S , Wang T , Zhu L , Torchilin VP . Hypoxia‐targeted siRNA delivery. Angew Chem Int Ed Engl. 2014;53(13):3362‐3366. doi:10.1002/anie.201308368 24554550 PMC4150469

[17] Ye Y , Hu Q , Chen H , et al. Characterization of hypoxia‐associated molecular features to aid hypoxia‐targeted therapy. Nat Metab. 2019;1(4):431‐444. doi:10.1038/s42255-019-0045-8 31984309 PMC6980239

[18] Zhuang Y , Liu K , He Q , Gu X , Jiang C , Wu J . Hypoxia signaling in cancer: implications for therapeutic interventions. MedComm. 2023;4(1):e203. doi:10.1002/mco2.203 36703877 PMC9870816

[19] Lee SH , Moroz E , Castagner B , Leroux JC . Activatable cell penetrating peptide–peptide nucleic acid conjugate via reduction of azobenzene PEG chains. J Am Chem Soc. 2014;136(37):12868‐12871. doi:10.1021/ja507547w 25185512

[20] Piao W , Tsuda S , Tanaka Y , et al. Development of azo‐based fluorescent probes to detect different levels of hypoxia. Angew Chem Int Ed Engl. 2013;52(49):13028‐13032. doi:10.1002/anie.201305784 24127124

[21] Kiyose K , Hanaoka K , Oushiki D , et al. Hypoxia‐sensitive fluorescent probes for in vivo real‐time fluorescence imaging of acute ischemia. J Am Chem Soc. 2010;132(45):15846‐15848. doi:10.1021/ja105937q 20979363

[22] Takakura Y , Takahashi Y . Strategies for persistent retention of macromolecules and nanoparticles in the blood circulation. J Control Release. 2022;350:486‐493. doi:10.1016/j.jconrel.2022.05.063 36029894

[23] van der Horst HJ , Nijhof IS , Mutis T , Chamuleau MED . Fc‐engineered antibodies with enhanced Fc‐effector function for the treatment of B‐cell malignancies. Cancers (Basel). 2020;12(10):3041. doi:10.3390/cancers12103041 33086644 PMC7603375

[24] Zhou F , Fu T , Huang Q , et al. Hypoxia‐activated PEGylated conditional aptamer/antibody for cancer imaging with improved specificity. J Am Chem Soc. 2019;141(46):18421‐18427. doi:10.1021/jacs.9b05063 31584808

[25] Hao D , Meng Q , Jiang B , et al. Hypoxia‐activated PEGylated paclitaxel prodrug nanoparticles for potentiated chemotherapy. ACS Nano. 2022;16(9):14693‐14702. doi:10.1021/acsnano.2c05341 36112532

[26] Qi S , Su L , Li J , et al. Arf6‐driven endocytic recycling of CD147 determines HCC malignant phenotypes. J Exp Clin Cancer Res. 2019;38(1):471. doi:10.1186/s13046-019-1464-9 31752956 PMC6868876

[27] Qi S , Su L , Li J , et al. YIPF2 is a novel Rab‐GDF that enhances HCC malignant phenotypes by facilitating CD147 endocytic recycle. Cell Death Dis. 2019;10(6):462. doi:10.1038/s41419-019-1709-8 31189879 PMC6561952

[28] Zhao P , Zhang W , Wang SJ , et al. HAb18G/CD147 promotes cell motility by regulating annexin II‐activated RhoA and Rac1 signaling pathways in hepatocellular carcinoma cells. Hepatology. 2011;54(6):2012‐2024. doi:10.1002/hep.24592 21809360

[29] Kuang YH , Chen X , Su J , et al. RNA interference targeting the CD147 induces apoptosis of multi‐drug resistant cancer cells related to XIAP depletion. Cancer Lett. 2009;276(2):189‐195. doi:10.1016/j.canlet.2008.11.010 19097686

[30] Pereira NA , Chan KF , Lin PC , Song Z . The “less‐is‐more” in therapeutic antibodies: afucosylated anti‐cancer antibodies with enhanced antibody‐dependent cellular cytotoxicity. MAbs. 2018;10(5):693‐711. doi:10.1080/19420862.2018.1466767 29733746 PMC6150623

[31] Wong CC , Kai AK , Ng IO . The impact of hypoxia in hepatocellular carcinoma metastasis. Front Med. 2014;8(1):33‐41. doi:10.1007/s11684-013-0301-3 24234682

[32] Liu J , Zhang Q , Chen H , et al. Phage display library selection of a hypoxia‐binding scFv antibody for liver cancer metabolic marker discovery. Oncotarget. 2016;7(25):38105‐38121. doi:10.18632/oncotarget.9460 27203546 PMC5122375

[33] Sharma A , Arambula JF , Koo S , et al. Hypoxia‐targeted drug delivery. Chem Soc Rev. 2019;48(3):771‐813. doi:10.1039/c8cs00304a 30575832 PMC6361706

[34] Sun Z , Huang J , Su L , et al. Arf6‐mediated macropinocytosis‐enhanced suicide gene therapy of C16TAB‐condensed Tat/pDNA nanoparticles in ovarian cancer. Nanoscale. 2021;13(34):14538‐14551. doi:10.1039/d1nr03974a 34473182

[35] Ke X , Fei F , Chen Y , et al. Hypoxia upregulates CD147 through a combined effect of HIF‐1α and Sp1 to promote glycolysis and tumor progression in epithelial solid tumors. Carcinogenesis. 2012;33(8):1598‐1607. doi:10.1093/carcin/bgs196 22678117 PMC6276922

[36] Ross WC , Warwick GP . Reduction of cytotoxic azo compounds by hydrazine and by the xanthine oxidase–xanthine system. Nature. 1955;176(4476):298‐299. doi:10.1038/176298a0 13253554

[37] Niu X , Su L , Qi S , Gao Z , Zhang Q , Zhang S . GRP75 modulates oncogenic Dbl‐driven endocytosis derailed via the CHIP‐mediated ubiquitin degradation pathway. Cell Death Dis. 2018;9(10):971. doi:10.1038/s41419-018-1039-2 30250167 PMC6155137

[38] Piao W , Hanaoka K , Fujisawa T , et al. Development of an azo‐based photosensitizer activated under mild hypoxia for photodynamic therapy. J Am Chem Soc. 2017;139(39):13713‐13719. doi:10.1021/jacs.7b05019 28872304

[39] Verwilst P , Han J , Lee J , Mun S , Kang HG , Kim JS . Reconsidering azobenzene as a component of small‐molecule hypoxia‐mediated cancer drugs: a theranostic case study. Biomaterials. 2017;115:104‐114. doi:10.1016/j.biomaterials.2016.11.023 27886551

[40] Zhang Y , Zhao W , Chen Y , et al. Rational construction of a reversible arylazo‐based NIR probe for cycling hypoxia imaging in vivo. Nat Commun. 2021;12(1):2772. doi:10.1038/s41467-021-22855-0 33986258 PMC8119430

[41] Hu M , Yang C , Luo Y , et al. A hypoxia‐specific and mitochondria‐targeted anticancer theranostic agent with high selectivity for cancer cells. J Mater Chem B. 2018;6(16):2413‐2416. doi:10.1039/c8tb00546j 32254457

[42] Perche F , Biswas S , Patel NR , Torchilin VP . Hypoxia‐responsive copolymer for siRNA delivery. Methods Mol Biol. 2016;1372:139‐162. doi:10.1007/978-1-4939-3148-4_12 26530922

[43] Peng S , Ouyang B , Xin Y , et al. Hypoxia‐degradable and long‐circulating zwitterionic phosphorylcholine‐based nanogel for enhanced tumor drug delivery. Acta Pharm Sin B. 2021;11(2):560‐571. doi:10.1016/j.apsb.2020.08.012 33643831 PMC7893141

[44] Zhu R , Baumann RP , Penketh PG , Shyam K , Sartorelli AC . Hypoxia‐selective O6‐alkylguanine‐DNA alkyltransferase inhibitors: design, synthesis, and evaluation of 6‐(benzyloxy)‐2‐(aryldiazenyl)‐9H‐purines as prodrugs of O6‐benzylguanine. J Med Chem. 2013;56(3):1355‐1359. doi:10.1021/jm301804p 23311288 PMC3722860

[45] van Brakel R , Vulders RC , Bokdam RJ , Grüll H , Robillard MS . A doxorubicin prodrug activated by the Staudinger reaction. Bioconjug Chem. 2008;19(3):714‐718. doi:10.1021/bc700394s 18271515

[46] Li S , Jiang X , Zheng R , et al. An azobenzene‐based heteromeric prodrug for hypoxia‐activated chemotherapy by regulating subcellular localization. Chem Commun (Camb). 2018;54(57):7983‐7986. doi:10.1039/c8cc03430c 29963672

[47] Wang Y , Xiao D , Li J , et al. From prodrug to pro‐prodrug: hypoxia‐sensitive antibody–drug conjugates. Signal Transduct Target Ther. 2022;7(1):20. doi:10.1038/s41392-021-00833-8 35058439 PMC8776858

[48] Li Q , White JB , Peterson NC , et al. Tumor uptake of pegylated diabodies: balancing systemic clearance and vascular transport. J Controlled Release. 2018;279:126‐135. doi:10.1016/j.jconrel.2018.04.013 29653224

[49] Yamamoto M , Kurino T , Matsuda R , et al. Delivery of aPD‐L1 antibody to i.p. tumors via direct penetration by i.p. route: beyond EPR effect. J Controlled Release. 2022;352:328‐337. doi:10.1016/j.jconrel.2022.10.032 36280153

[50] Klassen DK . Evidence for both oxygen and non‐oxygen dependent mechanisms of antibody sensitized target cell lysis by human monocytes. Blood. 1980;56(6):985‐992. 7437519

[51] Balsamo M , Manzini C , Pietra G , et al. Hypoxia downregulates the expression of activating receptors involved in NK‐cell‐mediated target cell killing without affecting ADCC. Eur J Immunol. 2013;43(10):2756‐2764. doi:10.1002/eji.201343448 23913266

[52] Zatovicova M , Kajanova I , Barathova M , et al. Novel humanized monoclonal antibodies for targeting hypoxic human tumors via two distinct extracellular domains of carbonic anhydrase IX. Cancer Metab. 2022;10(1):3. doi:10.1186/s40170-022-00279-8 35109923 PMC8811981

[53] Chang DK , Moniz RJ , Xu Z , et al. Human anti‐CAIX antibodies mediate immune cell inhibition of renal cell carcinoma in vitro and in a humanized mouse model in vivo. Mol Cancer. 2015;14:119. doi:10.1186/s12943-015-0384-3 26062742 PMC4464115

[54] Baysal H , De Pauw I , Zaryouh H , et al. Cetuximab‐induced natural killer cell cytotoxicity in head and neck squamous cell carcinoma cell lines: investigation of the role of cetuximab sensitivity and HPV status. Br J Cancer. 2020;123(5):752‐761. doi:10.1038/s41416-020-0934-3 32541873 PMC7462851

[55] Solocinski K , Padget MR , Fabian KP , et al. Overcoming hypoxia‐induced functional suppression of NK cells. J Immunother Cancer. 2020;8(1):e000246. doi:10.1136/jitc-2019-000246 32345623 PMC7213912

[56] Colombani T , Rogers ZJ , Bhatt K , et al. Hypoxia‐inducing cryogels uncover key cancer‐immune cell interactions in an oxygen‐deficient tumor microenvironment. Bioactive Mater. 2023;29:279‐295. doi:10.1016/j.bioactmat.2023.06.021 PMC10432785 37600932

[57] Cardarelli PM , Rao‐Naik C , Chen S , et al. A nonfucosylated human antibody to CD19 with potent B‐cell depletive activity for therapy of B‐cell malignancies. Cancer Immunol Immunother. 2010;59(2):257‐265. doi:10.1007/s00262-009-0746-z 19657637 PMC11030752

[58] Zhang S , Xing J , Zhang Q , et al. Optimal design of Ig 5' primers for construction of diverse phage antibody library established to select anti‐HAb18GEF and anti‐DOTA‐Y Fabs for hepatoma pretargeting RIT. Front Biosci. 2006;11:1733‐1749. doi:10.2741/1919 16368552

[59] Marcatili P , Rosi A , Tramontano A . PIGS: automatic prediction of antibody structures. Bioinformatics. 2008;24(17):1953‐1954. doi:10.1093/bioinformatics/btn341 18641403

[60] Lee LS , Conover C , Shi C , Whitlow M , Filpula D . Prolonged circulating lives of single‐chain Fv proteins conjugated with polyethylene glycol: a comparison of conjugation chemistries and compounds. Bioconjug Chem. 1999;10(6):973‐981. doi:10.1021/bc990076o 10563766

[61] Connolly ML . Solvent‐accessible surfaces of proteins and nucleic acids. Science. 1983;221(4612):709‐713. doi:10.1126/science.6879170 6879170

[62] Liang Y , Li X , Peng F , et al. Self‐assembly of X‐shaped antibody to combine the activity of IgG and IgA for enhanced tumor killing. Theranostics. 2022;12(18):7729‐7744. doi:10.7150/thno.74903 36451853 PMC9706586

[63] Tsao LC , Crosby EJ , Trotter TN , et al. Trastuzumab/pertuzumab combination therapy stimulates antitumor responses through complement‐dependent cytotoxicity and phagocytosis. JCI Insight. 2022;7(6):e155636. doi:10.1172/jci.insight.155636 35167491 PMC8986081

[64] Su L , Sun Z , Qi F , et al. GRP75‐driven, cell‐cycle‐dependent macropinocytosis of Tat/pDNA‐Ca(2+) nanoparticles underlies distinct gene therapy effect in ovarian cancer. J Nanobiotechnol. 2022;20(1):340. doi:10.1186/s12951-022-01530-6 PMC9301890 35858873

[65] Li J , Qi F , Su H , et al. GRP75‐faciliated mitochondria‐associated ER membrane (MAM) integrity controls cisplatin‐resistance in ovarian cancer patients. Int J Biol Sci. 2022;18(7):2914‐2931. doi:10.7150/ijbs.71571 35541901 PMC9066115

