# Supplementary material for: Hypoxia‐activated ADCC‐enhanced humanized anti‐CD147 antibody for liver cancer imaging and targeted therapy with improved selectivity
Source: MedComm (2020). 2024 Mar 11;5(3):e512. doi: 10.1002/mco2.512 (PMC10927247; doi:10.1002/mco2.512)
Supplement: Supplementary file 1 — Supporting Information [file MCO2-5-e512-s001.docx]

**Supplementary**

**Hypoxia-activated ADCC-enhanced humanized anti-CD147 antibody for liver cancer imaging and targeted therapy with improved selectivity**

Fang-Zheng Qi^1#^, Hui-Shan Su^1#^, Bo Wang^1#^, Luo-Meng Qian^1^, Yang Wang^1^, Chen-Hui Wang^1^, Ya-Xin Hou^1^, Ping Chen^2^, Qing Zhang^2^, Dong-Mei Li^3^, Hao Tang^4^, Jian-Li Jiang^4^, Hui-Jie Bian^4^, Zhi-Nan Chen^4^, Si-He Zhang^1^*

^1^ Department of Cell Biology, School of Medicine, Nankai University, Tianjin, 300071, China.

^2^ National Clinical Research Center for Cancer, Tianjin Medical University Cancer Institute and Hospital, Tianjin, 300060, China.

^3^ State Key Laboratory of Medicinal Chemical Biology, College of Pharmacy and Tianjin Key Laboratory of Molecular Drug Research, Nankai University, Tianjin 300353, China.

^4^ National Translational Science Center for Molecular Medicine, Department of Cell Biology, State Key Laboratory of Cancer Biology, Air Force Medical University, Xi'an, 710032, China.

^#^ These authors contributed equally.

***Correspondence:** Prof. Si-He Zhang,

Department of Cell Biology, School of Medicine, Nankai University

94 Weijin Road, Nankai District, Tianjin, 300071, China

E-mail: sihezhang@nankai.edu.cn

<https://orcid.org/0000-0002-8923-1993>

**Materials and Methods**

**1. Other antibodies, kits and chemicals**

Mouse anti-β-actin Ab (KM9001) and goat anti-human IgG (H+L)-HRP (LK2005) were obtained from Sungene Biotech. Goat anti-human IgG (H+L)-rhodamine Red (109-295-003) was obtained from Jackson. Goat anti-mouse IgG (H+L)-AF488 (A-11001) and a BCA protein assay kit (23225) were obtained from Life. Human IgG (SP001), Ficoll (P4360), RBC lysis buffer (R1010), and PMSF (329-98-6) were obtained from Solarbio. 5-amino-1-pentanol (A67299) was obtained from Innochem; N-bromosuccinimide (NBS; 128-08-5), Hoechst 33342 (B2261), gelatin (G1890) and other chemicals were obtained from Sigma.

**2. Lymphocyte isolation**

PBMCs were separated with lymphocyte separation medium. The cells were centrifuged for 30 minutes, collected, resuspended, and treated with RBC lysis buffer for 15 minutes. After cell-spinning, the PBS-resuspended cells were laid over Ficoll and centrifuged for 45 minutes. The top layer, which mostly contained the monocytes, was collected.

**3. Synthesis of amine-reactive PEG_5000_-azo-NHS ester**

PEG_5000_-azo-NHS ester was synthesized as described.[^1^](#_ENREF_1)^,^[^2^](#_ENREF_2) Some modifications were made to the synthesis process by Thinkerytech Technology Co., Ltd. (Tianjin, China). PEG_5000_-azo-NHS ester (compound #7) was produced by coupling PEG_5000_-azo (compound #6) with N,N'-disuccinimidyl carbonate (DSC). ^1^H NMR spectra were conducted on a Bruker Av400 (Bruker, Switzerland).

**3.1 Synthesis of compound #2**

MPEG_5000_ (5.0 g, 1.0 mmol) was dissolved in THF (100 mL) followed by adding NaOH solution (320.0 mg, 8.0 mmol). P-toluenesulfonyl chloride (381.3 mg, 2.0 mmol), dissolved in THF (5 mL), was added dropwise into the mixture on ice bath. Finally, the mixture was stirred until the end of the reaction was reached at RT, as indicated by thin layer chromatography. After the insoluble matter was removed, the filtrate was enriched under reducing pressure. The obtained residue was purified by silica gel column chromatography (methanol/DCM) to give compound #2 (PEG_5000_-tosylate, 3.4 g, 65% yield).

^1^H NMR (400 MHz, CDCl_3_) δ 7.81 (d, J = 8.3 Hz, 2H), 7.36 (d, J = 8.0 Hz, 2H), 4.19-4.15 (m, 2H), 3.99-3.45 (m, 255H), 3.39 (s, 3H), 2.46 (s, 3H).

**3.2 Synthesis of compound #5**

4-Aminobenzyl alcohol (615.4 mg, 5.0 mmol) was water-dissolved (10 mL), followed by adding 1.04 mL of concentrated HCl. NaNO_2_ (362.3 mg, 5.25 mmol, in 2.5 mL H_2_O) was added to the mixture, which was cold kept. After one hour of stirring, phenol (493.7 mg, 5.25 mmol) in 10% Na_2_CO_3_ (988.1 mg, 7.15 mmol, in 8.0 mL H_2_O) was added for 10 min reaction, and the resulting mixture was stirred for another three hours at RT. The final pH of mixture was adjusted to 7.2 with carbonate, and the resulting solids were filtered, washed and yielded the compound #5 (741.3 mg, 65% yield).

^1^H NMR (400 MHz, DMSO-d6) δ 10.31 (s, 1H), 7.79 (dd, J = 8.6, 1.8 Hz, 4H), 7.49 (d, J = 8.4 Hz, 2H), 6.94 (d, J = 8.8 Hz, 2H), 5.37 (t, J = 5.4 Hz, 1H), 4.59 (d, J = 4.3 Hz, 2H).

**3.3 Synthesis of compound #6**

Compound #5 (136.8 mg, 0.6 mmol) and K_2_CO_3_ (829.3 mg, 6.0 mmol) were DMF-dissolved (30 mL). Then, compound #2 (1.5 g, 0.3 mmol) in DMF (20 mL) was added, and the mixture was stirred at 50 °C for 12 hours. After the solvent was removed under reduced pressure, the crude product was purified by the silica gel column chromatography to yield the compound #6 (1.6 g, 50% yield).

^1^H NMR (400 MHz, CDCl_3_) δ 7.88 (dd, J = 12.6, 8.6 Hz, 4H), 7.50 (d, J = 8.2 Hz, 2H), 7.03 (d, J = 8.9 Hz, 2H), 4.77 (d, J = 5.8 Hz, 2H), 4.25-4.19 (m, 2H), 3.64 (s, 490H), 3.38 (s, 3H).

**3.4 Synthesis of PEG_5000_-azo-NHS ester (compound #7)**

Compound #6 (1 g, 0.2 mmol) was dissolved in dry toluene (20 mL), and the solvent was removed under reduced pressure. The procedure was repeated three times to remove water traces from compound #6. The compound was subsequently dissolved in dry DCM (30 mL). N,N'-Disuccinimidyl carbonate (DSC, 153.6 mg, 0.6 mmol) was added, followed by adding triethylamine (TEA, 0.6 mmol). The mixture was stirred overnight at RT. The crude product was purified with a preequilibrated PD10 column to produce the compound #7 as a yellow solid (749.2 mg, 70% yield).

^1^H NMR (400 MHz, CDCl_3_) δ 7.96-7.89 (m, 4H), 7.54 (d, J = 8.5 Hz, 2H), 7.05 (d, J = 9.0 Hz, 2H), 5.40 (t, J = 5.8 Hz, 2H), 4.27-4.19 (m, 2H), 3.99-3.40 (m, 349H), 3.39 (s, 3H), 2.87 (s, 4H).

**4. Pharmacokinetic analysis in mice**

The mice were routinely tested for rodent pathogens and found to be free of these pathogens. Mice (n=4/group) were intravenously injected with 5 or 25 mg/kg Abs in PBS. Blood samples were collected at 2, 4, 8, and 12 hours and on days 1, 2, 3, 4, 5, 7, 9 and 11 postinjection. The concentrations of the Abs in the serum were determined via ELISA. Briefly, individual wells of a 96-well plate (half-well) were coated with CD147 Ag (10 μg/mL). The plates were blocked with 3% BSA, incubated with serially diluted blood samples or standards (HcHAb18 and HAP18 Ab), and then incubated with an anti-human IgG (H+L) HRP conjugate (1:2000). Peroxidase activity was detected with TMB substrate, and the absorbance at 450 nm was measured using a plate reader.

**5. Western blotting**

Ab-treated liver cancer cells were lysed in RIPA buffer. The samples were quantified by a BCA kit, resolved by 10% SDS‒PAGE, transferred onto a PVDF membrane, blotted with mouse anti-CD147 Ab (HAb18, 1:2000), anti-β-actin Ab (1:4000), anti-HIF-1a Ab (1:500), rabbit anti-PARP Ab (recognized both forms, 1:5000), anti-pro-Caspase-3 Ab (1:500), anti-pro-Caspase 9 Ab (1:500), anti-cleaved Caspase-3 Ab (1:500), and anti-cleaved Caspase-9 Ab (1:500), and finally detected with the corresponding HRP-labeled secondary Abs.

**6. Ab affinity checked by SPR**

The CM5 biosensor chips were captured with human CD147 Ag (20 µg/ml, sodium acetate buffer, pH 5.0) on a sample rack (immobilized with buffer containing EDC, NHS and ethanolamine). After a couple of running procedures, the KDs were monitored by injecting serial dilutions of the Abs (flow rate: 20 μL/min, 120 seconds for binding, and 300 seconds for dissociation). After each cycle, the chip was regenerated by using 50% DMSO at a flow rate of 20 μL/min for 30 seconds. The binding affinity (Kd) was calculated by fitting the binding data to a kinetic binding model (1:1) in Biacore T200 (GE).

**7. Ag binding and IFN-γ release examined by ELISA**

Human CD147 Ag-coated 96-well plates were washed with PBST, blocked with PBS (containing 10% nonfat milk) and incubated with the indicated concentrations of different Abs for one hour at 37°C. After thorough washing, goat anti-human IgG (H+L)-HRP solution was added and incubated for one hour, after which the TMB substrate was added for the reaction. The absorbance (450 nm) was read with microplate reader. PBMCs (containing NK cells) were cocultured with liver cancer cells and stimulated with Ab or Ab alone for 24 h. The supernatants were harvested, and soluble IFN-γ was analyzed according to the kit instructions.

**8. Cellular binding checked by flow cytometry and confocal imaging**

EDTA-detached cells were incubated with cold medium containing Abs for one hour. After washing with PBS, rabbit anti-human IgG (H+L)-RDM was added for one hour, followed by washing with PBS and counting by flow cytometry. The data were analyzed with FlowJo.

The attached cells were incubated with Ab-containing serum-free medium at 37 °C, rinsed twice with PBS/NaCl (1 M), fixed with 4% PFA, blocked with FBS, followed by staining with anti-human IgG(H+L)-RDM and Hoechst 33342 overnight at 4°C. Cells binding under hypoxia and normoxia was imaged by an Olympus FV1000 confocal microscope (excitation: 652 nm, emission: 674 nm). During the confocal imaging process, 30 cells were recorded in each group. In flow cytometry measurements, 10,000 cells were recorded per sample.

**9. Multicellular spheroids binding assay**

Agarose solution (1.5%, in DMEM) was autoclaved, aliquoted and cold stored. After redissolution by heating, the wells were coated with agarose solution followed by sterilization and solidification with UV irradiation. The cell suspension was added to agarose-coated wells and cultured for forming MCTSs. To evaluate the binding of Abs, normoxia- or hypoxia-cultured MCTSs were exposed to Cy7-labeled Abs (IgG as a control, 400 µM) overnight. After washing with PBST buffer, the fluorescence from MCTSs was collected under confocal microscopy (excitation: 755 nm, emission: 787 nm) via Z-stack imaging at 20 µm intervals. At least eight MCTSs were determined for each group.

**10. *In vitro* cytotoxicity, growth inhibition and apoptosis assays**

Liver cancer cells (5x10^3^/well) were grown under normoxic and hypoxic conditions, and starved first in serum-free medium overnight. Distinct Abs (5 or 25 µg/mL) were added for 24 hours of incubation, and cell viability was analyzed by using a CCK-8 kit. To evaluate growth inhibition, cells grown logarithmically were cultured under normoxia or hypoxia with the indicated concentrations of Abs for 24 hours. Cell growth inhibition was estimated by a CCK-8 Kit. Hoechst 33342 was used to stain the apoptotic cells. Abs-treated cells were fixed with 1% PFA for 30 min, washed with PBS, and observed under a fluorescence microscope. Apoptotic cells were characterized by morphological alterations, such as condensed nuclei and cell shrinkage. Cellular apoptosis and death were further determined by using Annexin V/7-AAD kits according to the instructions.

**11. Gelatin zymography assay**

For single-culture, 24 hours cultured medium (serum-free) was harvested from 3T3 or PBMCs or liver cancer cells treated with different dosage (5, 25 µg/mL) of Abs, and then 40 folds concentrated by using Amicon Ultra 10 k devices. Equivalent amounts of concentrated culture medium (CCM) were loaded in an SDS‒PAGE gel containing 1% gelatin, washed with 2.5% Triton, incubated with incubation buffer, and finally stained with 0.05% Coomassie blue. The levels of activated MMP2 and MMP9 were estimated in the unstained areas. For coculture, liver cancer cells were incubated first with different Abs, after which 3T3 cells or PBMCs were added at a density ratio of 1:3, after which the activated MMP was determined as described above.

**12. Cell migration and invasion assays**

Scratch-migration assay: Cells were grown in 24-well plates (5×10^4^, 0.6% gelatin coated) and serum-starved overnight. After the cell monolayer was scraped with a sterile micropipette tip, complete medium (10% FBS) with or without Abs was added (t=0). After incubating under normoxia or hypoxia for 24 or 48 hours, the cells were photographed under a phase-contrast microscope. The wounded area was measured and calculated by using ImageJ software (https://imagej.en.softonic.com/).

Matrigel-coated transwell invasion assay: Cells were starved overnight, harvested by trypsinization, and reseeded (5×10^4^) with Abs-containing serum-free medium in Matrigel-coated transwell inserts (8-μm pore size). The lower chamber was filled with 10% FBS medium. After incubating under normoxia or hypoxia for 24 and 48 hours, cells on the filter in the upper chamber were removed with a cotton swab, and cells on the underside were stained with a crystal violet solution and counted under a microscope.

**References**

1. Zhou F, Fu T, Huang Q, et al. Hypoxia-Activated PEGylated Conditional Aptamer/Antibody for Cancer Imaging with Improved Specificity. *J Am Chem Soc*. Nov 20 2019;141(46):18421-18427. doi:10.1021/jacs.9b05063

2. Lee SH, Moroz E, Castagner B, Leroux JC. Activatable cell penetrating peptide-peptide nucleic acid conjugate via reduction of azobenzene PEG chains. *J Am Chem Soc*. Sep 17 2014;136(37):12868-71. doi:10.1021/ja507547w


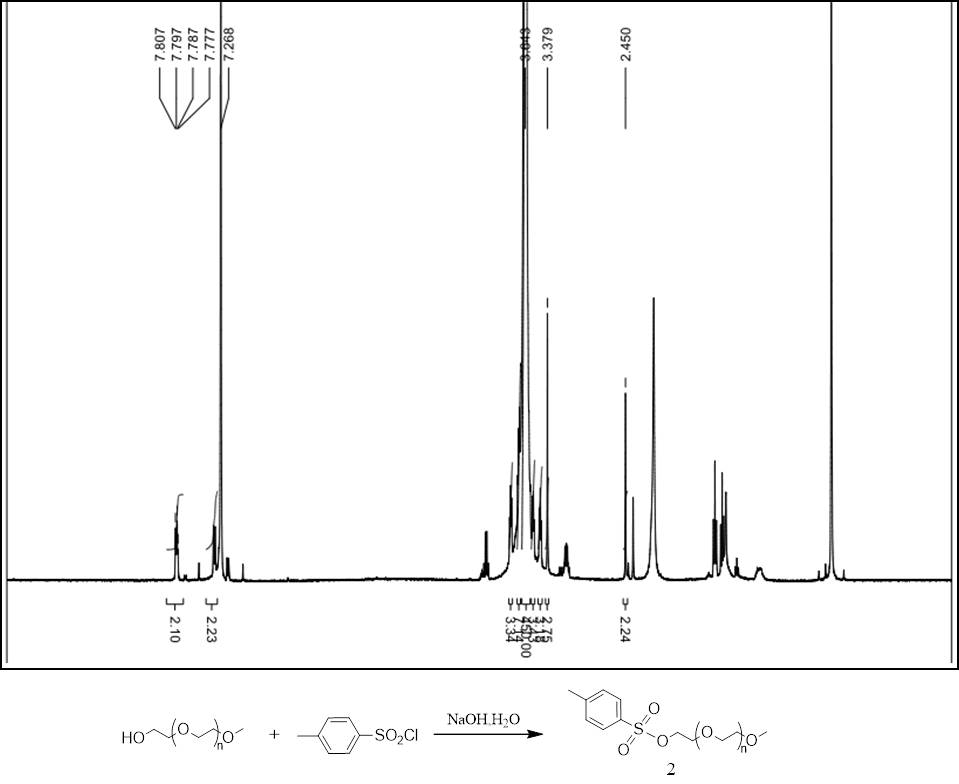


**Figure S1 ^1^H NMR spectra (δ, ppm; CDCl_3_, 400 MHz) of compound #2**. (doi:10.7150/ijbs.92211)


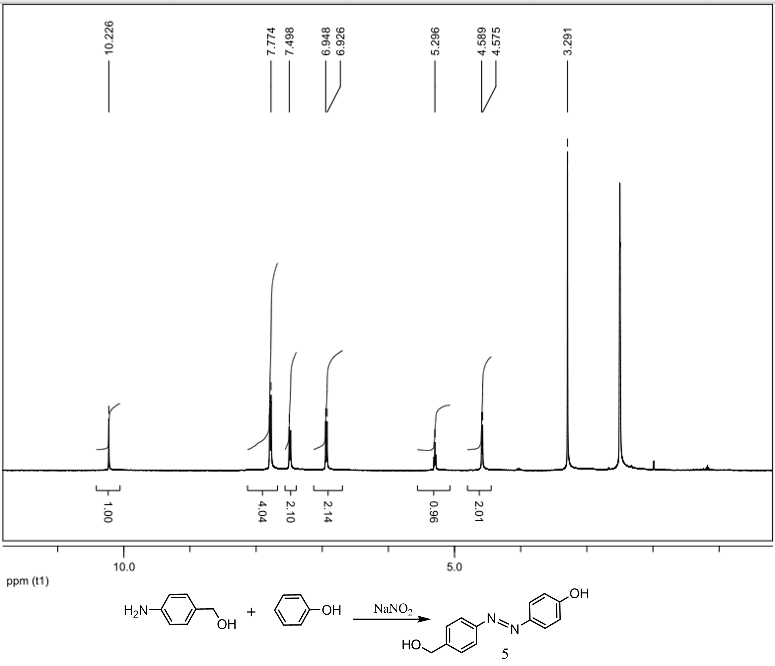


**Figure S2** **^1^H NMR spectra (δ, ppm; DMSO, 400 MHz) of compound #5.** (doi:10.7150/ijbs.92211)


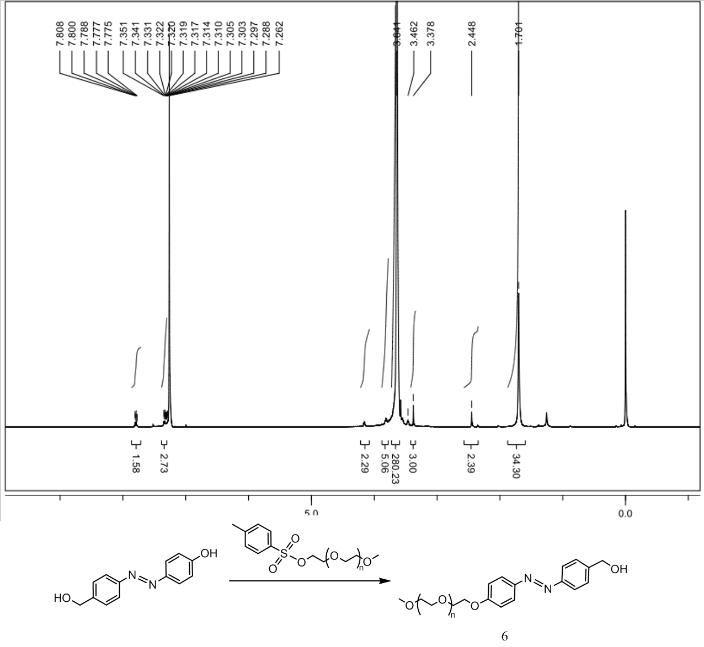


**Figure S3** **^1^H NMR spectra (δ, ppm; CDCl_3_, 400 MHz) of compound #6**. (doi:10.7150/ijbs.92211)


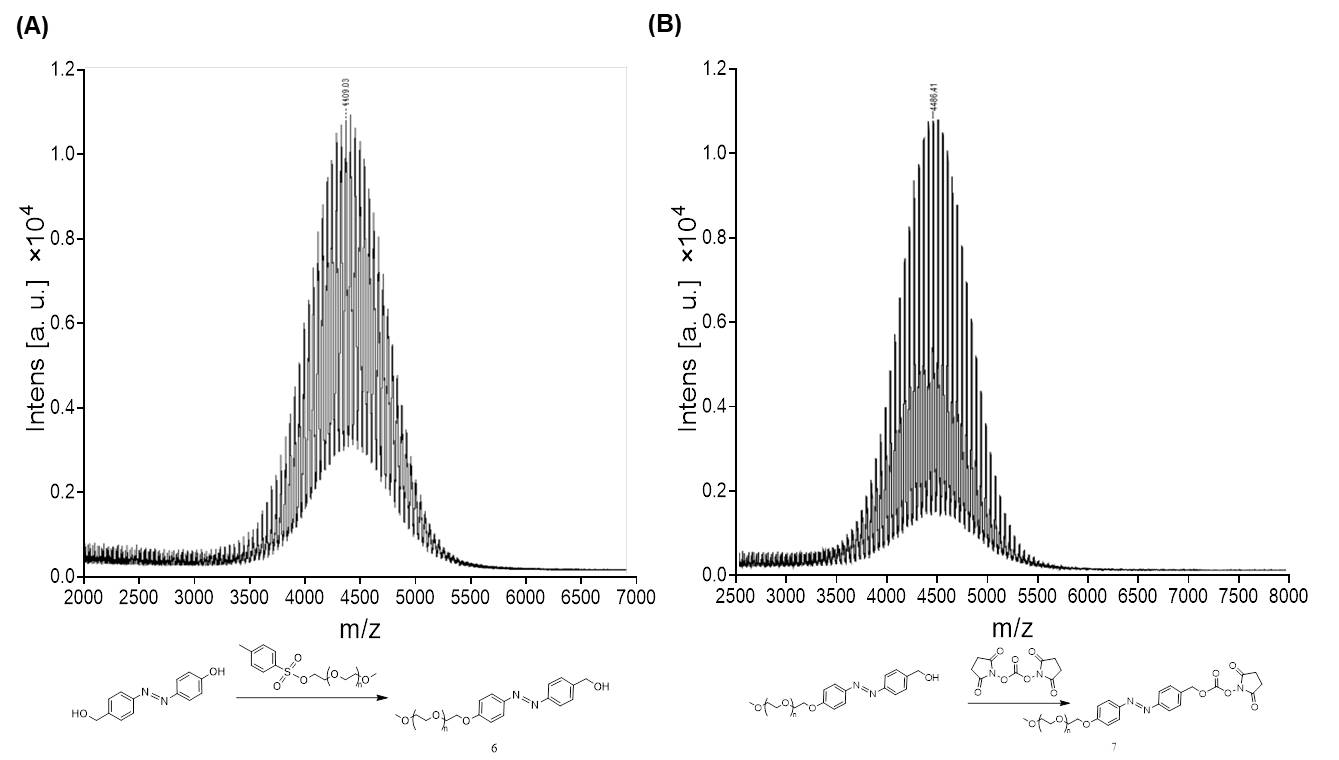


**Figure S4 Mass spectrum (MS) analysis of compound #6 (A) and compound #7 (B)**. (doi:10.7150/ijbs.92211)


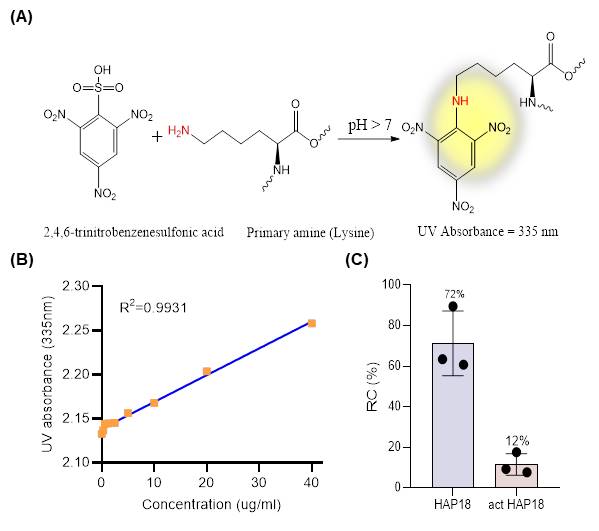


**Figure S5 Conjugation efficiency of the HAP18 Ab.** (**A**) Schematic illustration of the use of the TNBSA assay for quantifying the number of primary amino groups within Abs. (**B**) Calibration curve of the TNBSA assay. 5-Amino-1-pentanol was chosen as the standard regent. (**C**) Conjugation ratio (RC) of PEG_5000_-azo to HAP18 and the HAP18 Abs cleaved by Na_2_S_2_O_4_. The RC was defined as the percentage of PEG_5000_-azo conjugated to detectable amino groups. n=3.


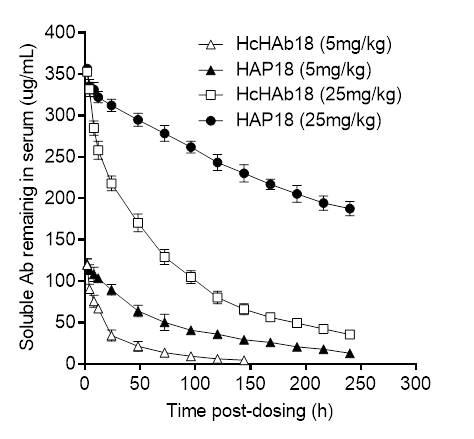


**Figure S6** **Pharmacokinetics of the Abs in mice**. Time course of the serum Ab concentration after a single i.v. administration (5 and 25 mg/kg).


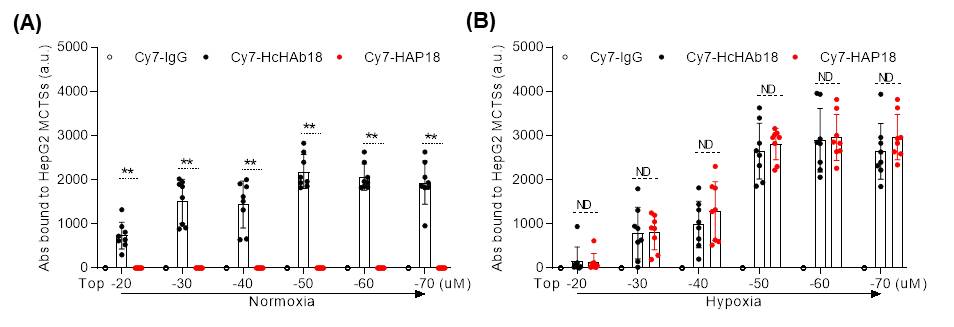


**Figure S7 Quantification of Abs bound to HepG2 MCTSs under normoxia (A) or hypoxia (B)**. Statistically significant differences compared with those of HcHAb18 are shown. n=8. Mean±SD, **P*<0.05, ***P*<0.01 vs. HcHAb18. ND: no significant difference. Student’s t test.


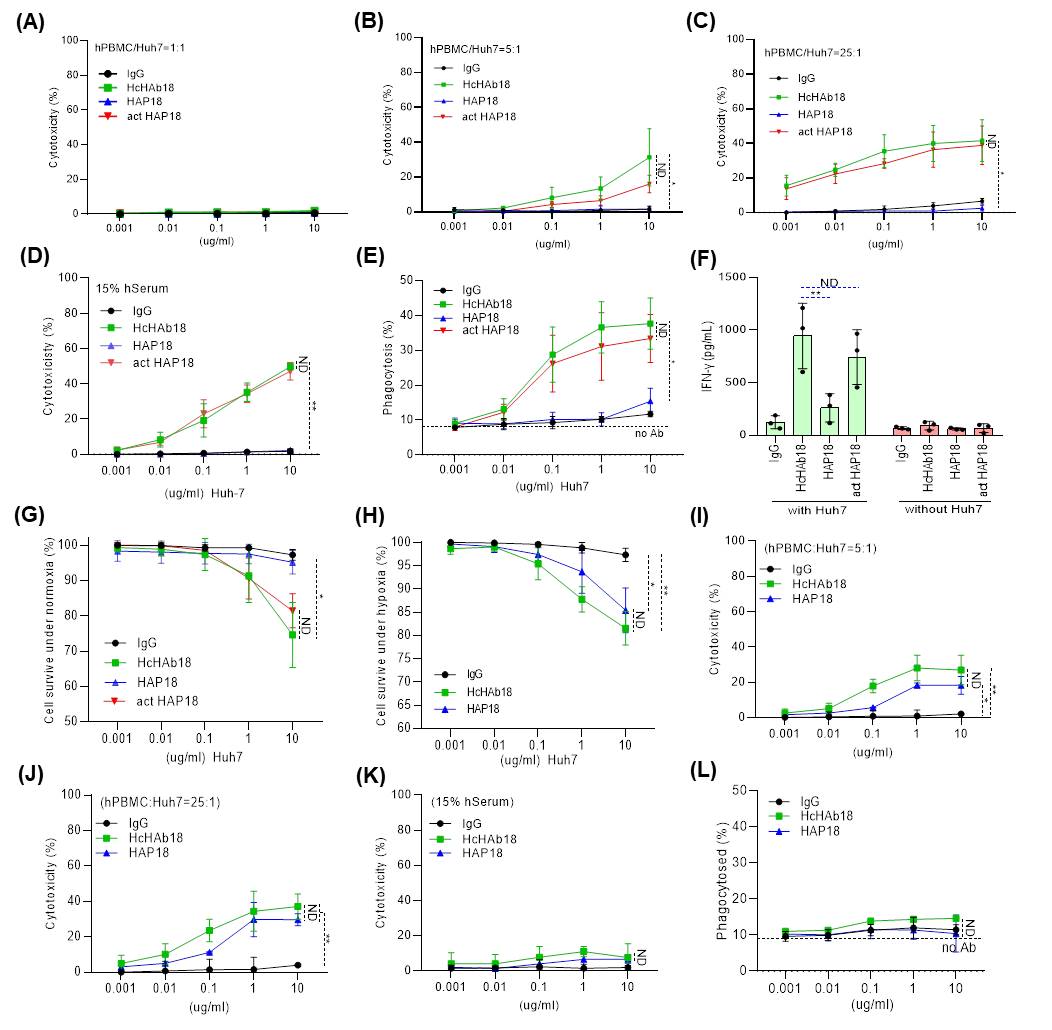


**Figure S8** ***In vitro* Ab-mediated immunological killing to Huh7 cells**. (**A**, **B**, **C**, **I**, **J**) ADCC activity mediated by Abs. PBMCs and Huh7 cells cocultured under normoxic (A, B, C) or hypoxic (I, J) conditions were assayed at different effector/target ratios (A, 1:1; B, I, 5:1; C, J, 25:1). (**D**, **K**) CDC activity mediated by Abs in Huh7 cells cocultured under normoxia (D) or hypoxia (K). (**E**, **L**) Abs-mediated ADCP activity in macrophages was tested by flow cytometry. Normoxic (E) or hypoxic (L) Huh7 cells were used as target cells. (**F**) IFN-γ was secreted from Ab-treated effector cells (PBMCs) with or without target cells (Huh7 cells). (**G**, **H**) The proliferation of normoxic (G) or hypoxic (H) Huh7 cells was inhibited by Abs. n=3. Mean±SD, **P*<0.05, ***P*<0.01 vs. IgG. ND: no significant difference. Two-way ANOVA.


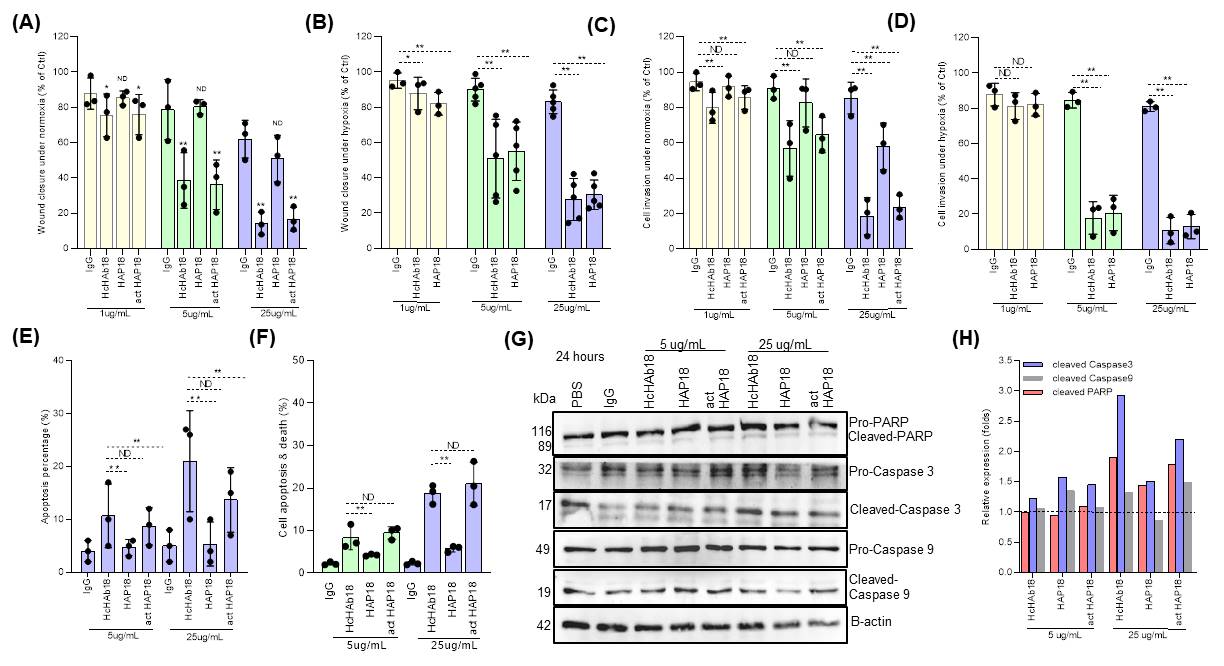


**Figure S9** **Quantification of cellular migration, invasion and apoptosis in Ab-treated HepG2 cells**. (**A, B**) Cellular migration. (**C, D**) Cellular invasion. (**E**) Hoechst 33342 staining. (**F**) Flow cytometry-based cellular apoptosis and death data. (**G, H**) Representative Western blots of pro- and activated PARP, Caspase-3 and Caspase-9 (F) and the corresponding quantification (G). n=3. Mean±SD, **P*<0.05, ***P*<0.01 vs. IgG or HcHAb18. ND: no significant difference. Two-way ANOVA.


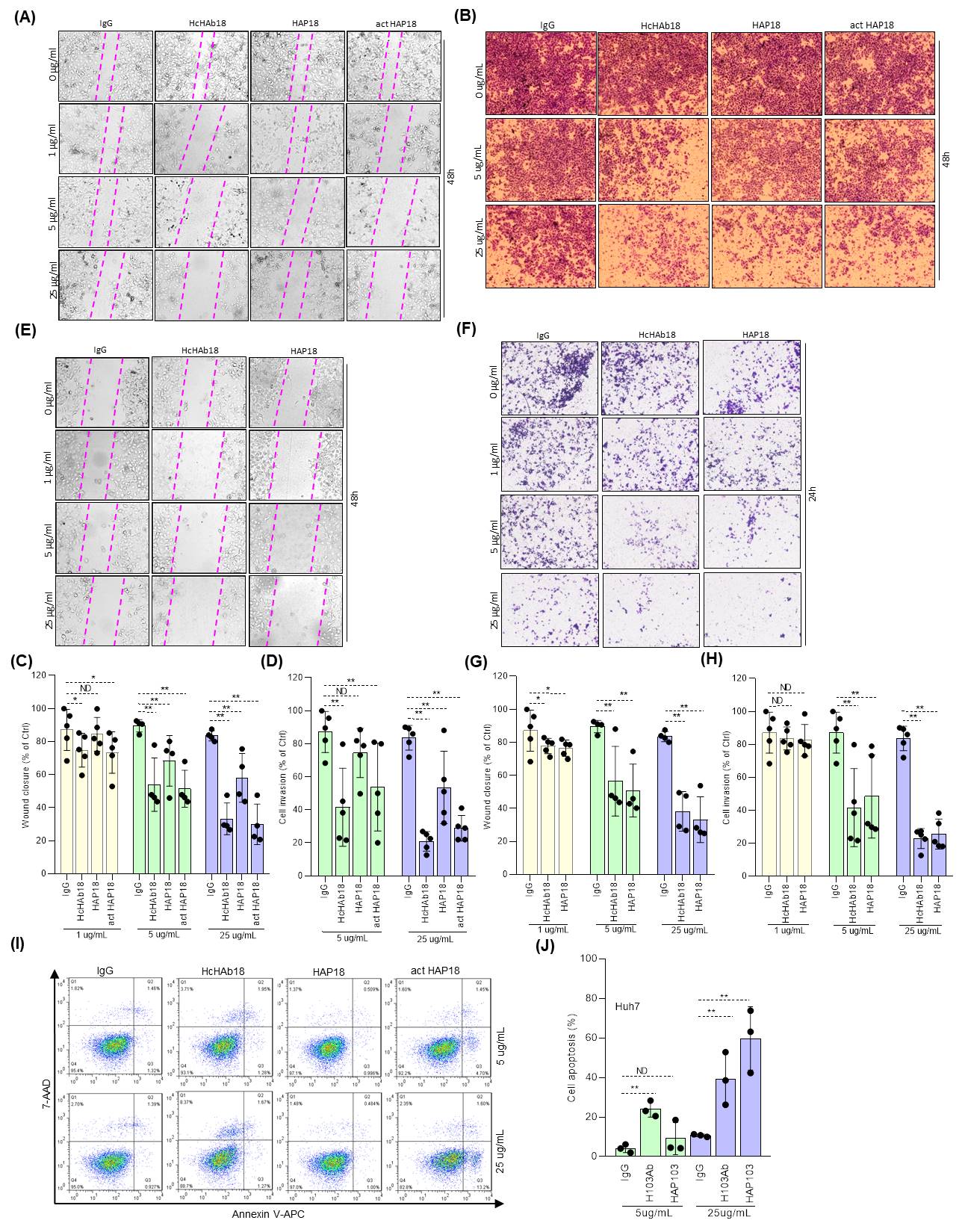


**Figure S10 *In vitro* Ab-induced changes in biological activity and apoptosis in Huh7 cells**. (**A**, **E**) Motility inhibition by different Abs in normoxic (A) or hypoxic (E) Huh7 cells was tested by scratch-migration assays. (**B**, **F**) Invasion inhibition by different Abs in normoxic (B) or hypoxic (F) Huh7 cells was tested by Matrigel-coated transwell assays. (**C**, **D, G**, **H**) Quantification of Huh7 cell migration (C, G) and invasion (D, H) affected by different Abs under normoxia (C, D) and hypoxia (G, H). (**I**, **J**) Representative flow cytometry-based apoptosis data for Huh7 cells and the corresponding quantification (J). n=3. Mean±SD, **P* < 0.05, ***P* < 0.01 vs. IgG. ND: no significant difference. Two-way ANOVA.


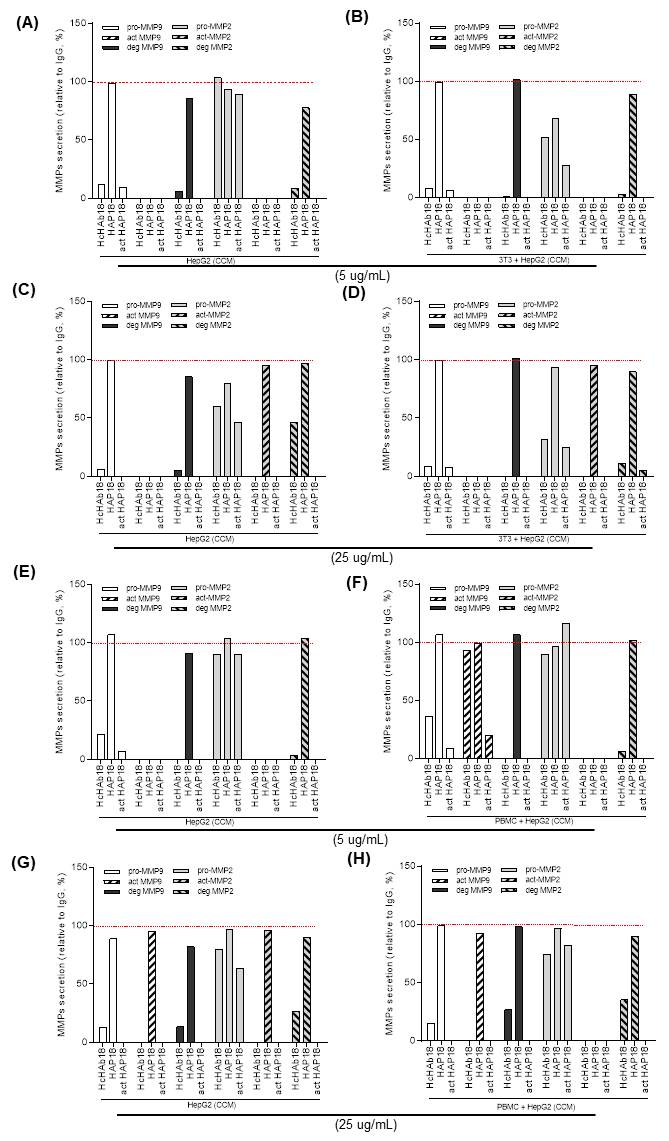


**Figure S11 Quantification of Ab-mediated inhibition of MMP activity in monocultures or cocultures** (corresponding to Fig. 5E-H)**.**

**
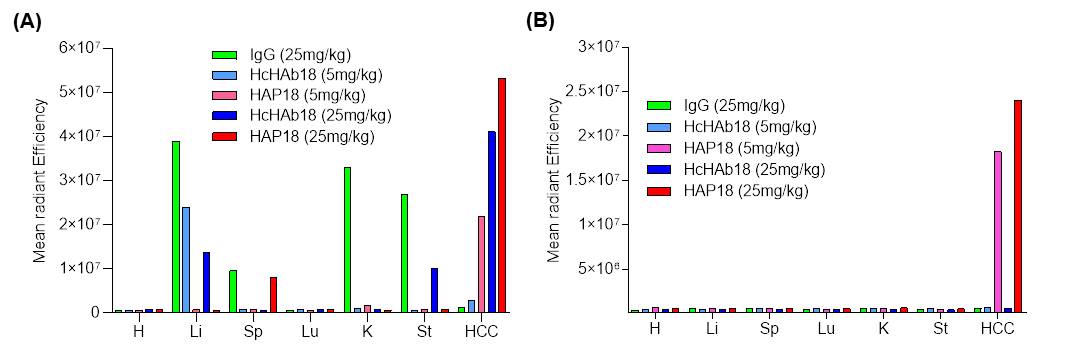
**

**Figure S12** **Quantification of the Cy7 fluorescence signal in organs and tumors** (corresponding to Fig. 6E&F).


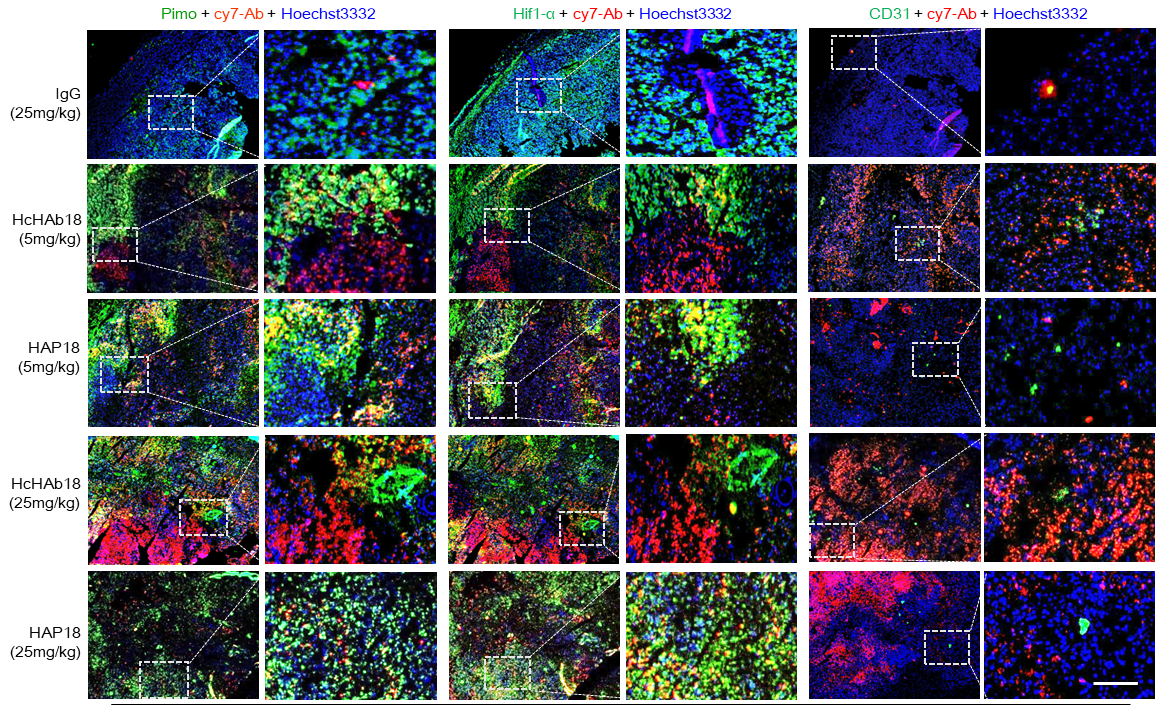


**Figure S13** **Multifluorescence analysis of Ab-targeting in human HCC-xenografted mice model**. Representative overlays of multiple digital fluorescence images of HepG2-formed HCC tissue from mice injected with Cy7-labeled Abs (red. Seventy-two hours before euthanization) and FITC-labeled pimonidazole (green. Thirty minutes before euthanization), or perfusion with Hoechst 33342 (blue. five minutes before euthanization), or blood vessel marker (green, anti-CD31 Ab staining). Scale bar=500 µm.


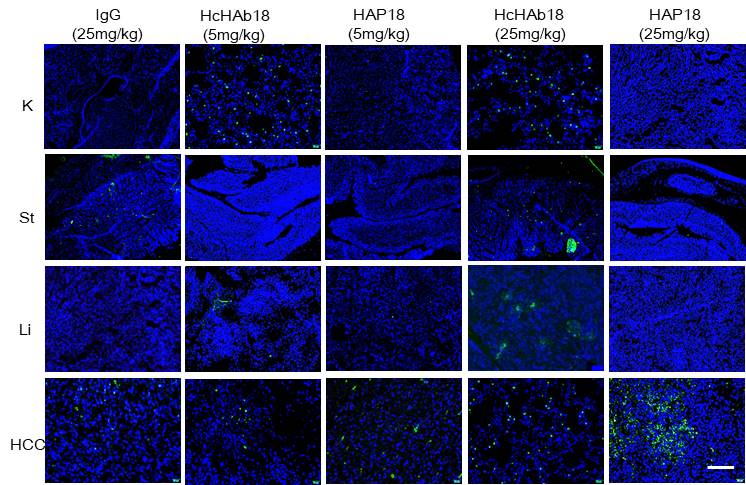


**Figure S14** **Apoptosis levels in organs and HCC tissues after Ab therapy.** Representative TUNEL staining signals (green) of the main organs and xenografted HCC tissues after challenge with different Abs.


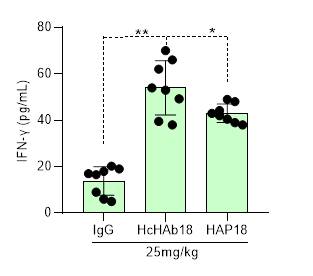


**Figure S15** **IFN-γ secretion after Ab therapy.** Mouse sera were collected at the end of treatment, and INF-γ levels were measured via ELISA. n=8. Mean±SD, **P* < 0.05, ***P* < 0.01 vs. HcHAb18. Student’s t test.


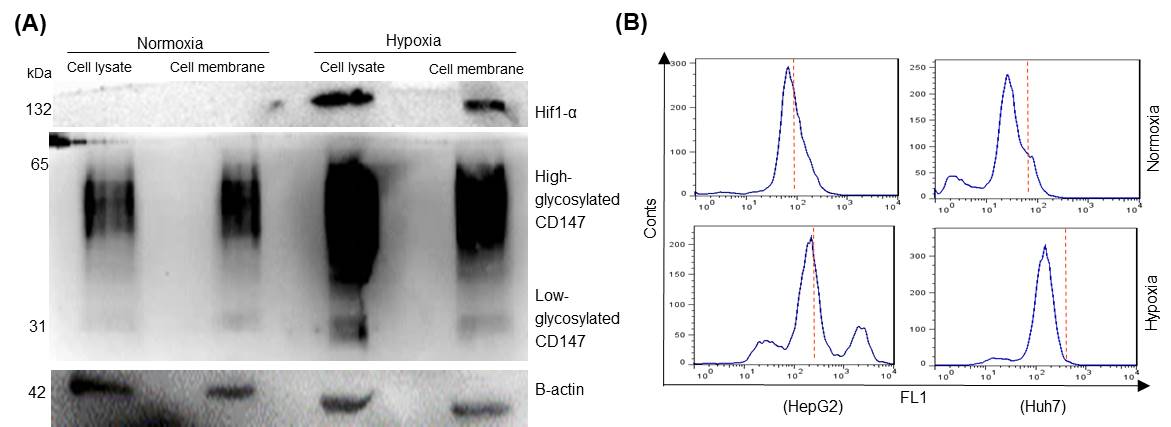


**Figure S16 CD147 expression in hypoxic and normoxic liver cancer cells**. (**A**) Western blot analysis of the CD147 expression level in HepG2 cell fractions. (**B**) Flow cytometry analysis of the expression level of CD147 on the HepG2 cell surface.


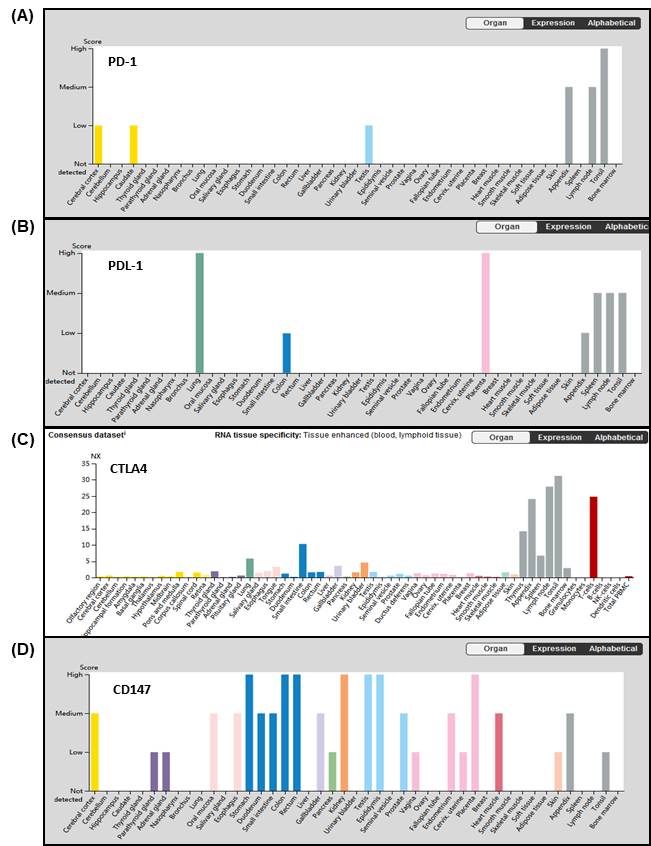


**Figure S17** **Expression profiles of PD-1 (A), PDL-1 (B), CTLA4 (C) and CD147 (D) in normal organs.** The data were retrieved from https://www.proteinatlas.org/.

**Table S1 Exposure pattern of lysine residues in the VH and VL of HcHAb18 Ab**.

| Residues | Solvent-accessible surface area | Connolly surface area | Surface-accessibility |
| --- | --- | --- | --- |
| VH |  | | |
| LYS3 | 33.7105 | 170.9733 | 19.72% |
| LYS19 | 3.1453 | 173.9171 | 1.81% |
| LYS43 | 70.2154 | 172.1242 | 40.79% |
| LYS54 | 92.5491 | 176.6027 | 52.41% |
| LYS67 | 57.2032 | 172.9439 | 33.08% |
| LYS78 | 54.5759 | 175.0444 | 31.18% |
| VL |  | | |
| LYS23 | 21.1657 | 175.2137 | 12.08% |
| LYS38 | -21.7799 | 177.3971 | -12.28% |
| LYS44 | 63.4588 | 177.1588 | 35.82% |
| LYS102 | 7.0128 | 175.0146 | 4.01% |
| LYS106 | 60.9889 | 174.5105 | 34.95% |

VH: heavy chain variable regions, VL: light chain variable regions;
